# Metabolome and Metagenome Integration Unveiled Synthesis Pathways of Novel Antioxidant Peptides in Fermented Lignocellulosic Biomass of Palm Kernel Meal

**DOI:** 10.3390/antiox13101253

**Published:** 2024-10-17

**Authors:** Hammad Qamar, Rong He, Yuanfei Li, Min Song, Dun Deng, Yiyan Cui, Miao Yu, Xianyong Ma

**Affiliations:** 1 Institute of Animal Science, Guangdong Academy of Agricultural Sciences, State Key Laboratory of Livestock and Poultry Breeding, Key Laboratory of Animal Nutrition and Feed Science in South China, Ministry of Agriculture and Rural Affairs, Guangdong Provincial Key Laboratory of Animal Breeding and Nutrition, Guangzhou 510640, China; drhammadqamar@gmail.com (H.Q.); herong4747@163.com (R.H.); song-min@gdaas.cn (M.S.); dengdun@gdaas.cn (D.D.); cuiyiyan@gdaas.cn (Y.C.); yumiao@gdaas.cn (M.Y.); 2 Institute of Biological Technology, Jiangxi Provincial Key Laboratory of Poultry Genetic Improvement, Nanchang Normal University, Nanchang 330032, China; li-yuan-fei@outlook.com; 3 Maoming Branch, Guangdong Laboratory for Lingnan Modern Agricultural, Maoming 525000, China

**Keywords:** enzymes, fermentation, metabolomics, metagenomics, antioxidants, *Lactobacillus*, palm kernel meal

## Abstract

Approximately one-third of the entire world’s food resources are deemed to be wasted. Palm kernel meal (PKM), a product that is extensively generated by the palm oil industry, exhibits a unique nutrient-rich composition. However, its recycling is seldom prioritized due to numerous factors. To evaluate the impact of enzymatic pretreatment and *Lactobacillus plantarum* and *Lactobacillus reuteri* fermentation upon the antioxidant activity of PKM, we implemented integrated metagenomics and metabolomics approaches. The substantially enhanced (*p* < 0.05) property of free radicals scavenging, as well as total flavonoids and polyphenols, demonstrated that the biotreated PKM exhibited superior antioxidant capacity. Non-targeted metabolomics disclosed that the *Lactobacillus* fermentation resulted in substantial (*p* < 0.05) biosynthesis of 25 unique antioxidant biopeptides, along with the increased (*p* < 0.05) enrichment ratio of the isoflavonoids and secondary metabolites biosynthesis pathways. The 16sRNA sequencing and correlation analysis revealed that *Limosilactobacillus reuteri*, *Pediococcus acidilactici*, *Lacticaseibacillus paracasei*, *Pediococcus pentosaceus*, *Lactiplantibacillus plantarum*, *Limosilactobacillus fermentum*, and polysaccharide lyases had significantly dominated (*p* < 0.05) proportions in PMEL, and these bacterial species were strongly (*p* < 0.05) positively interrelated with antioxidants peptides. Fermented PKM improves nutritional value by enhancing beneficial probiotics, enzymes, and antioxidants and minimizing anti-nutritional factors, rendering it an invaluable feed ingredient and gut health promoter for animals, multifunctional food elements, or as an ingredient in sustainable plant-based diets for human utilization, and functioning as a culture substrate in the food sector.

## 1. Introduction

Processing foods generates around 33% of the worldwide wasted food, therefore giving rise to substantial economic, environmental, and climatic concerns [1]. Across the world, extraction and utilization of primary vegetable oils have seen substantial growth, with palm oil emerging as the dominating processed plant-based edible oil [2]. Currently, palm oil accounts for roughly 33% of the worldwide need for vegetable oils. Its annual supply is expected to reach 80.19 million tons by 2024. Consequently, the edible oil sector is expected to generate around 10.87 million tons of palm kernel meal (PKM) in 2024 [3,4]. Overall, PKM is regarded as a major protein and energy resource with a composition of 91% dry matter, 13–22% crude protein, 16–34% crude fiber, 11% oil, and 5.5% ash [5]. Furthermore, it serves as a reservoir of certain macronutrients, including calcium and phosphorus [6,7]. The difficult-to-digest components consist of non-starch polysaccharides (NSP), including 3% water-insoluble glucoxylans, 3% arabinoxylans, 12% cellulose, and 78% β-mannan [8]. Although the PKM has significant potential for exploitation, it remains underused despite its affordability and widespread availability [7]. The current application of PKM is primarily limited to using it for organic fertilizer, feed for animals, or energy production using biogas or combustion [9]. Utilizing PKM as fertilizer is restricted by its high oil percentage, which may hinder soil aeration and microbial growth [10]. As animal feed, its substantial amount of fiber may reduce digestibility in monogastric species like chickens [11]. Energy production techniques such as generating biogas are obstructed by the complicated lignocellulosic structure, which challenges microbial digestion [8]. In addition, PKM is utilized for power and steam production or is discarded at specified dumps, contributing to environmental pollution and greenhouse gas emissions (0.25–0.5 tons of CH_4_ is estimated to be produced per ton of PKM discarded) [12,13]. Therefore, while these methods offer possibilities, they do not come without challenges, necessitating additional investigation for efficient and sustainable ways of disposing.

The extraction of vital nutrients from PKM exhibits a substantial challenge since nutrients and proteins are strongly embedded into cellulose and hemicellulose, i.e., mannans and arabinoxylans [14,15]. To surmount this difficulty, of all the available alternatives, biologically pretreatment processes stand out as a more environmentally sustainable solution [16]. The enzymatic approach for hydrolysis of PKM is preferred due to its moderate conditions for operation, substrate precision, and environmental sustainability. Contrary to chemical hydrolysis, enzymatic procedures run at lower temperatures and neutral pH levels, which assists in maintaining the nutritional integrity and bioactive characteristics of the resulting hydrolysates and avoids the formation of harmful by-products [16,17]. Enzymes including cellulases and hemicellulases especially target the cellulose and hemicellulose portions, enabling the targeted emission of bioactive peptides, amino acids, or fermentable carbohydrates without creating harmful compounds [18]. This method additionally improves the digestibility of PKM, making it ideal for use in feeds for animals by improving nutrient accessibility [11]. The enzymatic operation can be tailored and scaled up, providing a more sustainable and economically feasible strategy for PKM utilization in different industries.

The beneficial effects of *Lactobacillus* fermentation for augmenting the antioxidant properties of numerous agricultural industries’ secondary products are becoming more prevalent. The process of fermentation initiated by *Lactobacillus* spp. assists in the dispensation and formation of antioxidant compounds, for instance, flavonoids, isoflavonoids, and polyphenolic compounds, from plant-derived ingredients [19]. *Lactobacillus* fermentation improves antioxidant capability through enzyme-mediated hydrolysis, creating bioactive compounds and enhancing the activity of antioxidant enzymes. This procedure boosts the bioavailability of antioxidants and reduces chemical elements that inhibit the action of antioxidants, consequently boosting the overall antioxidant potential of the final fermentation product [20]. The *Lactobacillus* fermentation efficiently degrades anti-nutritional elements, like phytic acid and tannins, that naturally occur in PKM and improves the bioavailability of important nutrients, including amino acids and minerals [21]. The creation of lactic acid by lactic acid bacteria decreases the pH, hindering the growth of spoilage bacteria and enhancing the meal’s shelf life [22]. Furthermore, *Lactobacilli* fermentation may supplement PKM with health-promoting probiotics, enhancing the gut functioning of livestock [23]. This approach is ecologically friendly, demanding no chemical compounds and producing less waste than conventional chemical treatments, rendering it a desirable choice for sustainable processing of PKM [24].

The overall nutritional value of fermented food is primarily governed by the microbial population and their main and secondary products [25]. Modern sequencing research, “omics” technology, and the integration of microbiome and metabolomic approaches have been demonstrated to be excellent tools for undertaking comprehensive studies of microbial populations and analyzing the metabolites of fermented substrates [26]. Metabolomics enables the analysis of several individual metabolites simultaneously rather than in groupings. Metabolomics is a useful tool for assessing the different metabolites involved in PKM fermentation. These approaches seem to be effective in determining how the complete microbial population and microbial secondary metabolites affect the nutritional content and functional parameters of fermented foods [27,28].

Very little emphasis has been paid to reducing the anti-nutritional components and increasing the nutritive value of PKM using a mix of enzymatic degradation and fermentation by microbes. The objective of the proposed study is to assess how enzymatic treatment can boost *Lactobacilli* fermentation and improve the nutritive value and antioxidant properties of PKM, as well as to investigate the metabolic changes and mechanisms involved during the fermentation process.

## 2. Materials and Methods

### 2.1. Enzymes and Microbial Strains

Kangyuan Hezhong Biotech (Zibo, China) and Xihai Biotech (Xi’an, China) provided the commercially prepared probiotics, *Lactobacillus plantarum* and *Lactobacillus reuteri*, respectively. The bacterial strains used for fermenting substrate possessed 10^10^ CFU/g of concentration. The two commercial enzymes (pectinase and cellulase) were gratefully delivered by VTR Biotechnology (Zhuhai, China), having an activity of 750,000 U/g and 50,000 U/g, respectively. The normal dosage amounts for cellulase and pectinase were 0.5 g and 0.3 g per kilogram, respectively. The commercially available enzyme (xylanase) was acquired from Sunson Biotechnology Limited (Beijing, China), having an activity of 30,000 U/g, and the dosage rate was 0.3 g per kilogram. Pectinase was selected because it targets pectin and helps in breaking the plant cell wall structure, encouraging the accessibility of other enzymes to cellulose and hemicellulose [29]. PKM is enriched with cellulose, and cellulase is particularly successful at converting this cellulose into fermentable sugars [30]. Xylanase was chosen since it hydrolyzes hemicellulose, specifically xylan, which is an essential element of the PKM cell wall [31].

After weighing precisely, all enzymes were dissolved in 3 mL of deionized water with the aid of an oscillator for around 10 min. Following that, the centrifugation of the enzyme mixture was performed at 5000× *g* for 10 min, and PKM was hydrolyzed using supernatant in subsequent experiments.

### 2.2. Palm Kernel Meal Hydrolysis and Lactobacilli Fermentation

The PKM was bought from the local feed mill. Prior to the experiment, PKM was originally processed by crushing it using a centrifugal grinder (Ultra-speed grinder, ZX Instrument, Beijing, China) to decrease the particle size. After crushing, the material was passed using a 40-mesh screen, which functioned to separate the larger particles from the finer ones. The larger particles that failed to go through the sieve were taken apart, re-ground utilizing the same centrifugal crusher, and screened once again via the 40-mesh sieve to ensure uniformity in particle size. This process was continued until the particles were adequately homogeneous. Eventually, only the fine, unified fragments that passed easily across the 40-mesh sieve were chosen for further experimentation for better consistency in the following analyses and procedures.

The PKM was fermented in 150 mL conical flasks sealed with aluminum foil and butyl rubber corks. There were three treatments, namely, the control group, with PKM only (CON); PKM with enzymes only (PME); and PKM with enzymes and *Lactobacilli* bacteria (PMEL). The QC01, QC02, and QC03 denote quality control samples. The H1, H2, and H3 represent the CON group. The H8, H9, and H10 symbolize the PME group, and H30, H31, and H32 denote the PMEL treatment.

Each treatment was run with six replicates. The 10 g of PKM and 37 mL of distilled water were added to each flask. At the beginning of the trial, 3 mL enzyme solution was poured into both the PME and PMEL treatments, while 3 mL of distilled water was poured into the CON treatment only. All the flasks were then transferred onto a shaker that was set to operate at 50 °C and 150 rpm for 28 h. Following 28 h, 2 g of the *Lactobacilli* mixture (1 g each of *L. plantarum* and *L. reuteri*) was incorporated into the PMEL alone, and the shaker was once again utilized for a further 48 h. The commercial enzymes exhibit peak efficiency around 50 °C. This temperature guarantees that the enzymes retain their catalytic activity without being denatured, thereby increasing the conversion rate of the substrate into wanted products [32]. The duration of 28 h is preferred to allow appropriate time for the enzymes to act completely on the substrate, warranting complete hydrolysis with no excessive degradation or loss of functional elements [33]. A 48 h period is perfect because it offers enough time for the *Lactobacillus* to multiply and generate lactic acid and other secondary metabolites, which is crucial to the fermentation process [19].

For further examination, the PKM samples were procured and saved at −80 °C after the experiment.

### 2.3. Metabolome Analysis

The methods previously mentioned were used to perform the metabolome analysis [34,35,36]. In short, 50 mg of PKM specimen and 6 mm crushing beads were added to a 2 mL centrifugal tube. Metabolite extraction was carried out employing a 400 μL retrieval solution (methanol–water = 4:1 *v*/*v*) comprising 0.02 mg/mL of an internal reference compound (L-2-chlorophenylalanine). All the samples were ground for 6 min (−10 °C, 50 Hz) utilizing a Wonbio-96c frozen specimen grinding machine (Shanghai Wanbo Biotechnology, Shanghai, China). After that, they were purified for 30 min at a low temperature (5 °C, 40 kHz) utilizing ultrasonic purifying technology. The collected samples were then centrifuged for 15 min at 4 °C and 13,000× *g* after being held at −20 °C for 30 min. The supernatant was subsequently shifted to an infusion tube for the analysis of metabolites via the high-performance liquid chromatography-mass spectrometry (HPLC-MS) technique utilizing a ThermoFisher HPLC apparatus (Thermo Fisher Scientific Inc., Waltham, MA, USA). This HPLC system contained a particular ACQUITY HSS T3 column (100 mm × 2.1 mm, 1.8 μm; Waters, Milford, MA, USA). For the separation process, two mobile phases were utilized: one made of 0.1% formic acid in a solution of water and acetonitrile (95:5, *v*/*v*), and the other was developed of 0.1% formic acid in a combination of acetonitrile, isopropanol, and water (47.5:47.5, *v*/*v*). The system functioned at a flow rate of 0.40 mL per minute, with the column maintained at a constant temperature of 40 °C. Every specimen injection volume was 3 μL. The mass spectrometric data were acquired employing the same HPLC system, packed with an electrospray ionization (ESI) source that operated in positive as well as negative ion modes. The analysis governed a mass range of 70 to 1050 *m*/*z*, offering comprehensive identification of the compounds found in the sample.

The metabolites were recognized by exploring the HMDB database (http://www.hmdb.ca/ (accessed on 15 May 2024)) and the KEGG database (http://www.genome.jp/kegg (accessed on 15 May 2024)). The distinct metabolites between the two groups were tracked into their biological pathways via metabolite enrichment and pathways analysis following the KEGG database of metabolites (https://www.genome.jp/kegg/pathway.html (accessed on 25 May 2024)). The Python package “scipy.stats” (https://docs.scipy.org/doc/scipy/ (accessed on 25 June 2024)) was utilized to carry out the analysis of principal components (PCA) and orthogonal least partial squares discriminant assessment (OPLS-DA), the reliability of the model (assessed using a 7-cycle interactive validation process), and to carry out an enrichment assessment to identify the most pertinent biological processes for different treatments. The metabolites with a VIP value of more than 1 and a *p*-value of less than 0.05 were identified as substantially distinct metabolites employing the variable significance in the projection (VIP) derived from the OPLS-DA model and the *p*-value generated through the Student’s *t*-test.

### 2.4. Metagenome Analysis

Following the previously explained methodologies, the extraction of DNA, metagenome sequencing process, and functional interpretations were carried out for metagenomic analysis [37]. The PKM quantity utilized for extracting DNA was 1 mL. In short, the Mag-Bind^®^ Soil DNA Kit was utilized to acquire entire genomic DNA from PKM samples following the directions provided by the manufacturer (Omega Biotek, Norcross, GA, USA). The NEXTFLEX Rapid DNA-Seq (Bio Scientific, Austin, TX, USA) was used for library generation, and DNA sequencing was accomplished on Illumina NovaSeq 6000 (Illumina Inc., San Diego, CA, USA) following the producer’s directions (www.illumina.com (accessed on 28 May 2024)).

After eliminating adaptors from the paired-end Illumina reads, poor-quality reads (less than 50 bp in length, less than 20 quality points, or N bases) were eliminated using fastp [38] (https://github.com/OpenGene/fastp (accessed on 30 May 2024)). Megahit (v 1.1.2) was employed for assembling the metagenomics dataset [39]. The eventual assembly outcome was contigs having a length of ≥300 bp, which were later utilized to carry out gene identification and annotations. The prodigal program was utilized for the prediction of the open reading frames (ORFs) across each assembled contig [40] (https://github.com/hyattpd/Prodigal/ (accessed on 12 June 2024)). Utilizing CD-HIT, a non-redundant genetic catalog was developed [41] (http://www.bioinformatics.org/cd-hit/ (accessed on 15 June 2024)). By employing SOAPaligner, the top-quality reads were correlated to non-redundant genetic catalogs to determine gene proportions possessing 95% similarity [42] (https://github.com/ShujiaHuang/SOAPaligner (accessed on 22 June 2024)). The Diamond was implemented to align the selected sequences from the non-redundant (NR) gene catalog to the NR dataset for assessing the taxonomic annotations [43] (http://diamondsearch.org/forums/index.php (accessed on 22 June 2024)). The Diamond tool was employed [43] (http://diamondsearch.org/forums/index.php (accessed on 22 June 2024)) for performing the KEGG annotation via mapping on the KEGG database (https://www.genome.jp/kegg/annotation/ (accessed on 22 June 2024)). The carbohydrate-active enzyme (CAZy) annotation was performed utilizing hmmscan (https://www.ebi.ac.uk/Tools/hmmer/search/hmmscan (accessed on 22 June 2024)) by mapping on the CAZy database (http://www.cazy.org/ (accessed on 22 June 2024)).

### 2.5. Antioxidant Capacity of PKM and Polyphenols and Flavonoids Content

The ABTS and the DPPH methods were implemented to assess the overall antioxidant activity of PKM, and commercially available kits were utilized for the determination of the total flavonoid and polyphenol percentage under the manufacturer’s guidelines [44,45]. All the commercial kits were procured from Michy Bioengineering (Suzhou, China).

The antioxidant capability of the PKM sample was determined via the ABTS method by detecting the modification in absorption at 734 nm against the control substance Trolox. A 1 mL extracting reagent was added after weighing a 0.10 g PKM sample. Post ice-based homogenization, centrifugation of PKM samples was carried out for 10 min at 10,000× *g* and 4 °C. For further experiments, the fluid portion was collected.

To determine the specimen’s antioxidant capacity via the DPPH method, the measurement of absorption variation was taken at 515 nm against the control substance Trolox. After weighing 0.10 g of PKM, 1 mL of extracting reagent was introduced. After homogenizing the specimens on ice, they went through a centrifuge under a temperature of 4 °C at 10,000× *g* for 10 min. A supernatant was acquired for further assessment.

To determine the flavonoid percentage, the PKM samples were desiccated to a constant mass, grounded, and sifted through a 40-mesh screen. The 60% ethanol was poured into 0.05 g of PKM. The solution was subsequently blended at 60 °C for 2 h before being centrifuged at 25 °C for 10 min at 10,000× *g*. The resulting solution above the sediment was collected for further analysis. The flavonoid percentage was determined by finding the absorption value of the isolate from the specimen at 510 nm.

The overall polyphenol concentration of the PKM material was determined by homogenizing it after drying to a uniform weight and then screening it over a 40-mesh sieve. A precisely weighed 0.02 g PKM sample was mixed with 1 mL of the extracting solvent. At 60 °C, a two-hour agitation was performed, and the resultant mixture was subjected to a ten-minute centrifugal rotation with force of 10,000× *g* at 25 °C temperature. The liquid phase was extracted to do further tests. The total amount of phenol in this specimen was estimated via measurement of its absorption at 765 nm. All the above specimens were analyzed for absorption employing the microplate reader.

### 2.6. Statistical Analysis

In the present research, we employed statistical tools from the Python module “scipy.stats” (https://docs.scipy.org/doc/scipy/ (accessed on 25 June 2024)) for assessing microorganisms’ taxonomic classification, CAZymes, KEGG pathways, and metabolites of interest. The Mann–Whitney test was utilized to determine variances among groups. To evaluate the interactions between microbial species and compounds, we applied Spearman correlation modeling, considering solely correlations possessing an absolute value of |r| above 0.6 and a *p*-value below 0.05 as having significance. Significant variances were noted at *p* < 0.05, and to further regulate for false positives, we customized the *p*-values employing the false discovery rate (FDR), with the ultimate significance threshold established at an adjusted *p*-value of ≤0.05. The Gephi application was subsequently employed to visualize the correlation networks (https://gephi.org/ (accessed on 25 June 2024)).

## 3. Results

### 3.1. Multi-Statistical Analysis

**Figure 1:**
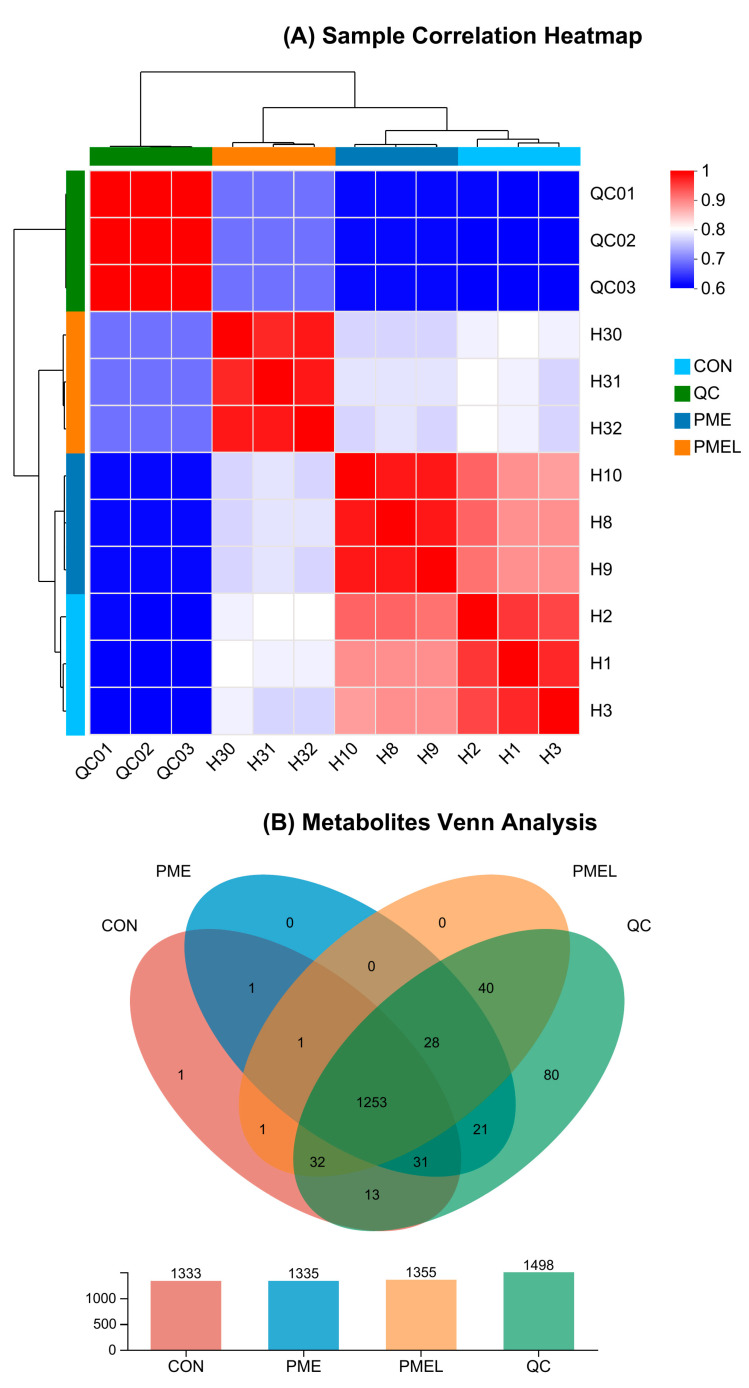

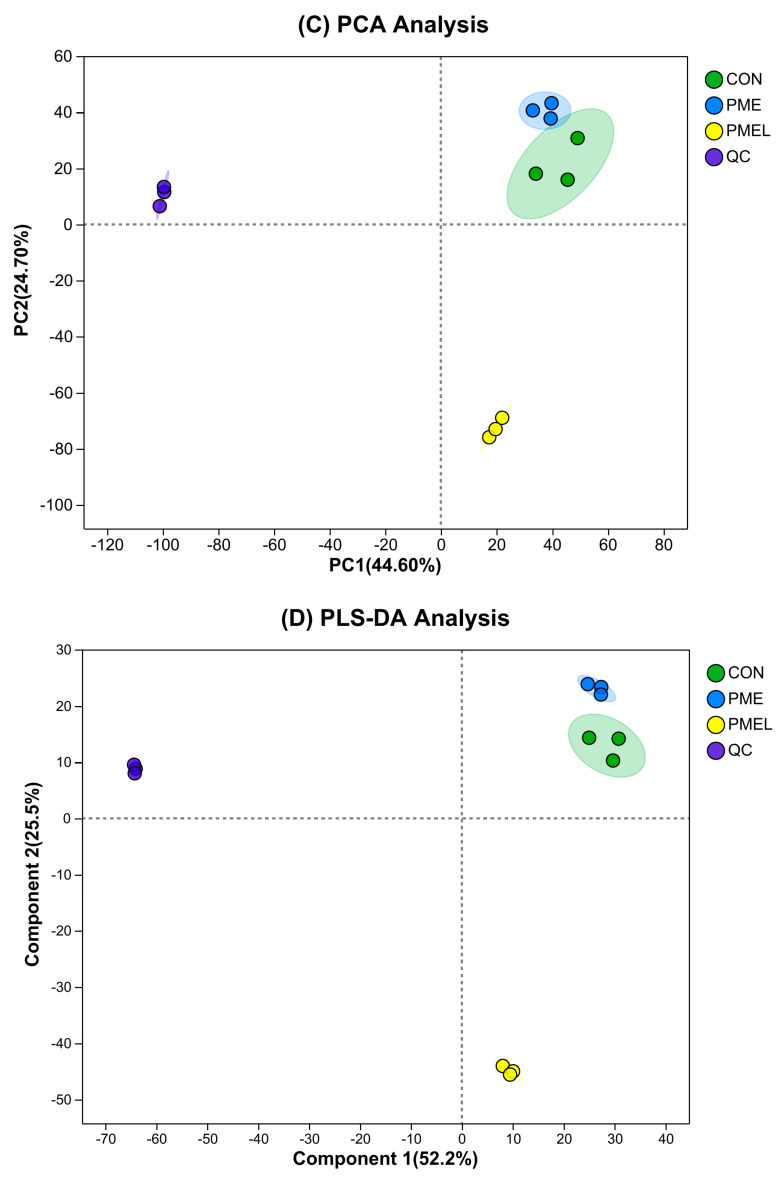

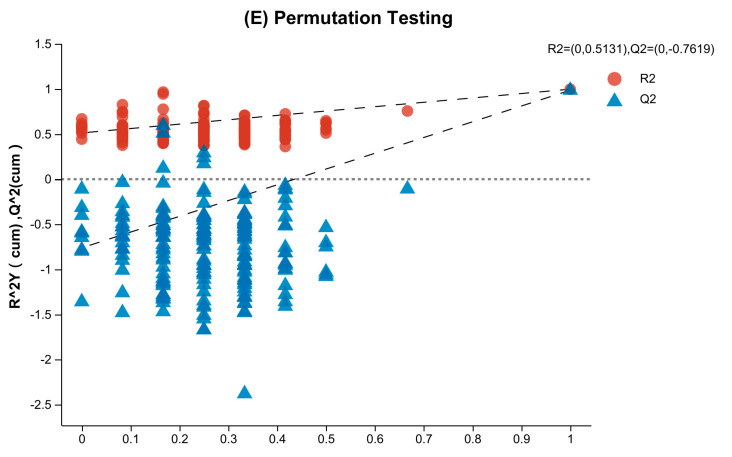
Samples Analyses: (**A**) Samples correlation heatmap analysis; (**B**) Metabolites Venn diagram analysis; (**C**) PCA assessment; (**D**) PLS-DA assessment; and (**E**) Permutation Testing of PLS-DA plot. Every box in the heatmap illustration represents the correlation between the two samples. The various hues correspond to the corresponding value of the correlation factor amongst samples, which ranged from 0.6 to 1. The different hues of Venn diagrams indicate distinct groupings. The Venn diagram’s sections that overlap show how many similar compounds there are in each group, while the non-overlapping sections show how many unique metabolites there are in each group, indicating variations in the groups’ metabolic profiles.

The coefficients related to the correlation of H30 with H31 and H32 were 0.97 and 0.98, correspondingly. The coefficients related to the correlation of H1 with H2 and H3 were 0.96 and 0.97, respectively. The CON samples showed a stronger correlation with the PME samples than the PMEL samples (Figure 1A). The PMEL asserted the greatest number of entire metabolites (1355), followed by PME (1335) and CON (1333) (Figure 1B).

Principal component (PC) 1 stipulated 44.60% of the variation, although PC 2 was accountable for 24.70% of the variation, according to the PCA scoring assessment. Collectively, PCs 1 and 2 had possession of 69.30% of the data’s fluctuation (Figure 1C). Moreover, the PLS-DA evaluation of the data demonstrated that Component 1 might explain 52.2% of the data deviation, whereas Component 2 was able to forecast 25.5% (Figure 1D). This proposed that there was an important distinction existing between CON and PMEL in metabolite diversity, which was additionally apparent from the PCA assessment. The Permutation Testing is designed to examine PLS-DA’s modeling and prediction performance. The R2 value of the PLS-DA plot was equivalent to 0.5131, and the upward trajectory of the regression line, coupled with reductions in R2 and Q2, revealed that the regression model did not appear overestimated and was trustworthy (Figure 1E).

### 3.2. Expression of Distinct Metabolites

**Figure 2:**
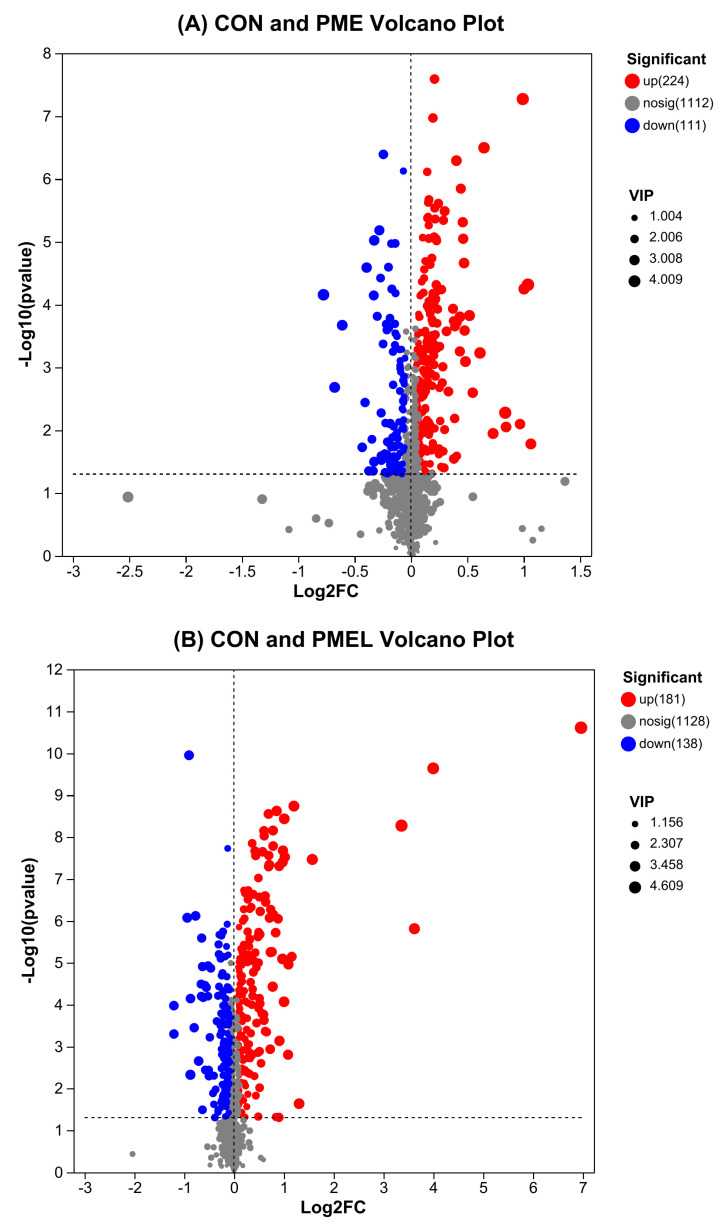

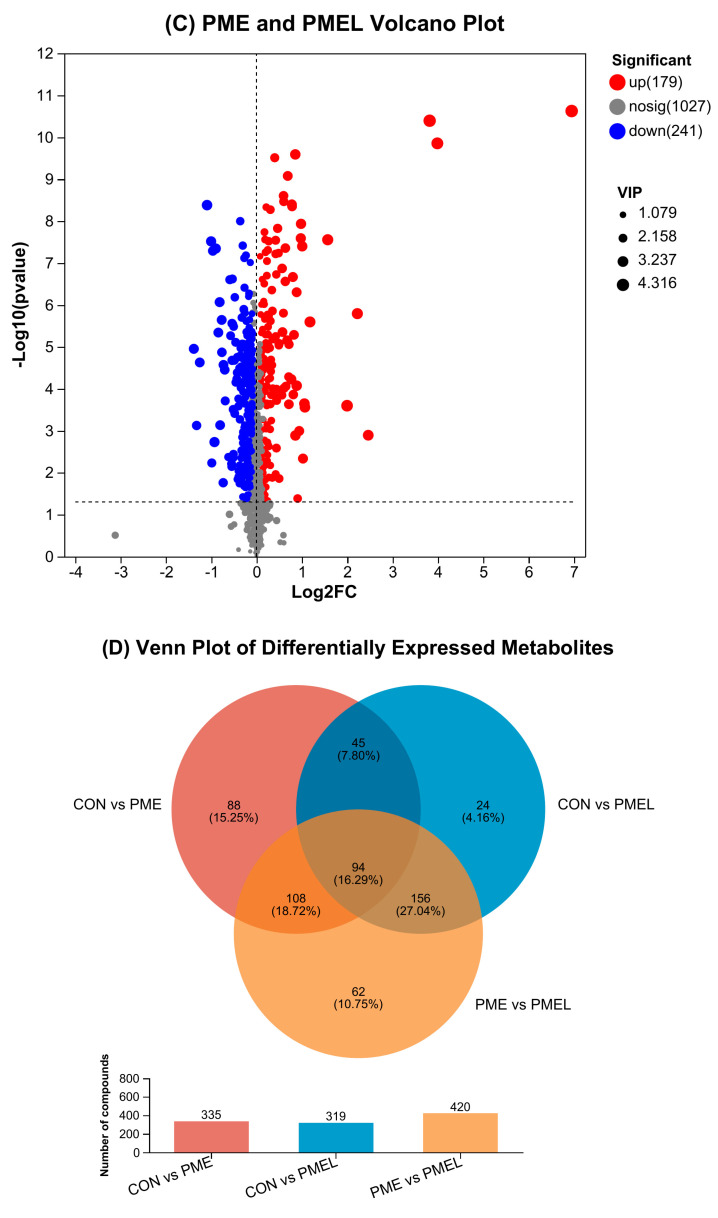
Summary of expression of distinct metabolites: (**A**) CON and PME Volcano plot. The top three upregulated metabolites in PME were trehalose, L-methionine, and glucoside. The top three downregulated metabolites in PME were glucosinolate, aminopicolinic acid, and salicylic acid; (**B**) CON and PMEL Volcano plot. The top three upregulated metabolites in PMEL were kaempferol 3-neohesperidin, tectorigenin, and kaempferol 3-neohesperidoside. The top three downregulated metabolites in PMEL were butyric acid, glyceric acid, and loperamide; (**C**) PME and PMEL Volcano plot. The top three upregulated metabolites in PMEL were kaempferol 3-neohesperidin, sesamolinol glucoside, and tectorigenin. The top three downregulated metabolites in PMEL were loperamide, tetraethylene glycol, and oxoundecylcarnitine, and (**D**) Venn plot of differentially expressed metabolites.

We established Volcano plots to assess the metabolites across different treatments. These plots enabled us to identify those molecules that were upregulated, downregulated or demonstrated no significant alteration between the groups. There were 224 upregulated and 111 downregulated metabolites detected in the PME group in comparison between CON and PME (Figure 2A). In comparison between CON and PMEL, 181 upregulated and 138 downregulated compounds were noticed in PMEL (Figure 2B). However, in comparison between PME and PMEL, increased and decreased metabolites were 179 and 241, respectively, in PMEL (Figure 2C). The Venn plot assessment of the differentially expressed metabolites (DEM) in the several groups being compared demonstrated that each group showcased distinct metabolites, indicating variations in metabolic profile among the various groups. There were 88, 24, and 62 distinct (DEM) detected in CON versus PME, CON vs. PMEL, and PME vs. PMEL, correspondingly (Figure 2D).

### 3.3. Compound Categorization in Accordance with KEGG and HMDB Databases

**Figure 3:**
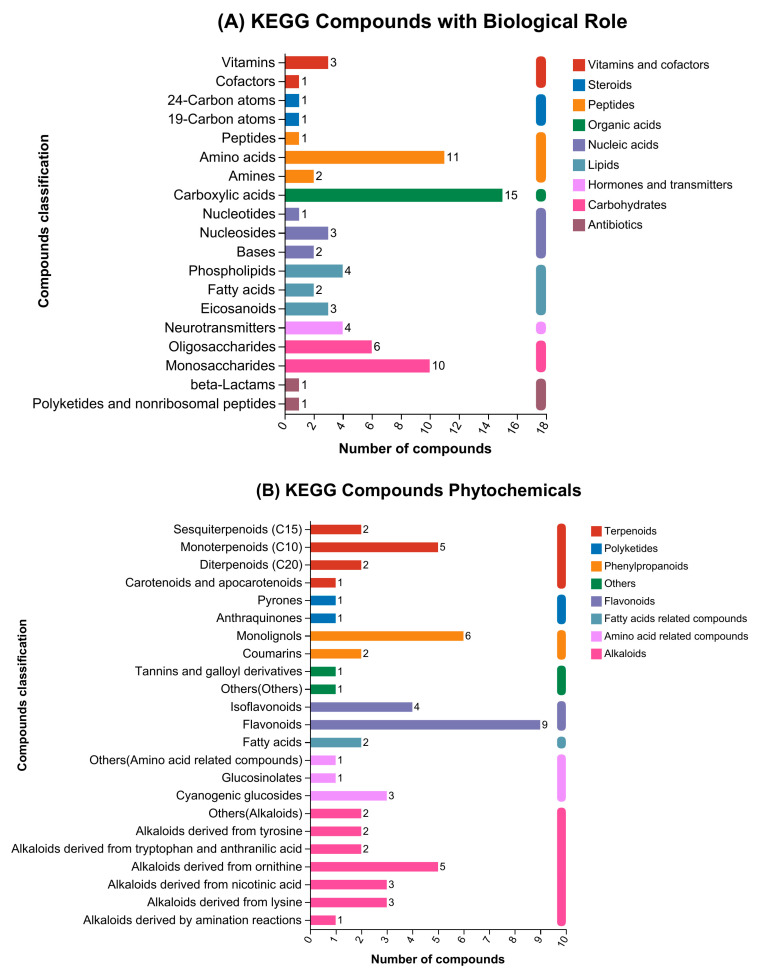

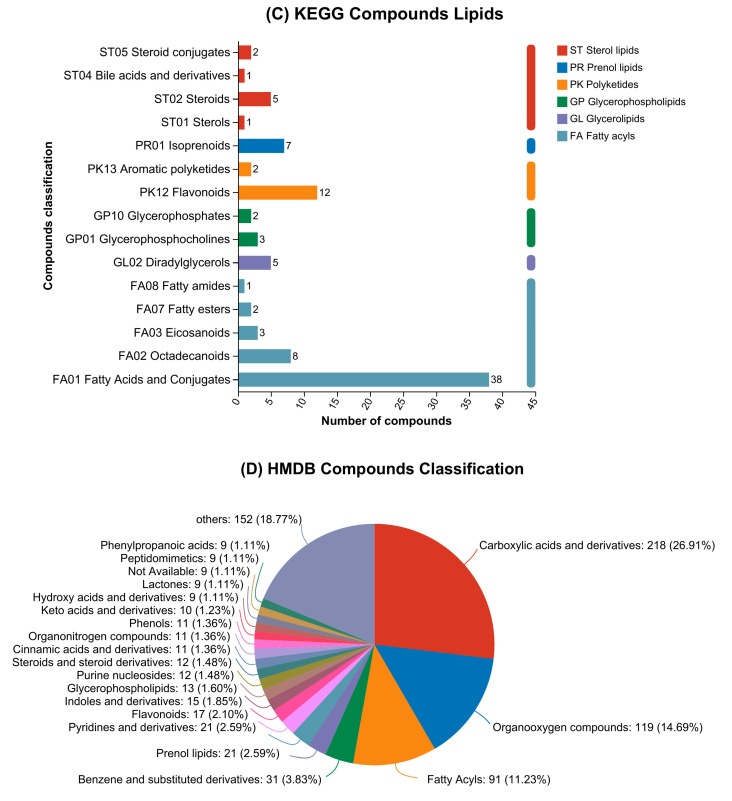

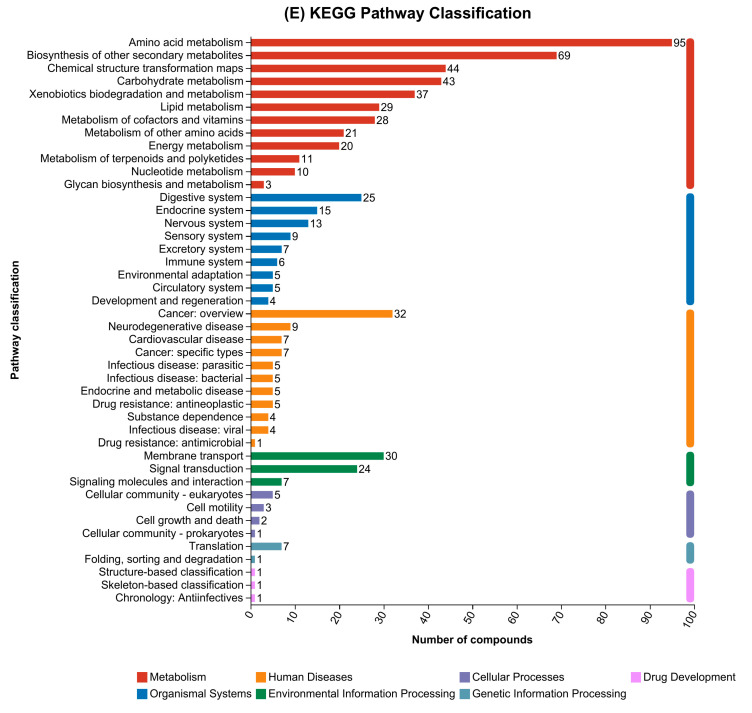
KEGG and HMDB compounds organization: (**A**) KEGG Compounds classification with biological role; (**B**) KEGG compounds classification of phytochemicals; (**C**) KEGG compounds classification of lipids; (**D**) HMDB compounds classification, and (**E**) KEGG compounds pathway classification.

To make categorization sense, the KEGG and HMDB indexes were employed for the categorizing of DEM. An aggregate of 224 metabolites was discovered based on the KEGG categorization. An aggregate of 72 metabolites was found conforming to the KEGG categorization of compounds having biological functions. The most varied and prevalent substances consisted of carbohydrates, which were followed by phospholipids, amino acids, monosaccharides, and oligosaccharides (Figure 3A). There were 60 biochemicals identified in the KEGG repository of phytochemical substances. Between them, flavonoids were the most varied and prevalent biomolecules, followed by monolignols, alkaloids, monoterpenoids, and isoflavonoids (Figure 3B). An aggregate of 92 metabolites was identified using the KEGG grouping of lipid substances. The most varied and prevalent substances were the conjugates and fatty acids, which were succeeded by steroids, flavonoids, octadecanoids, and isoprenoids (Figure 3C).

In accordance with the HMDB categorization of substances, 810 biochemicals were discovered. The most ample and diverse compounds were carboxylic acids and derivatives (26.91%), followed by organooxygen compounds (14.69%), fatty acyls (11.23%), benzene and substituted derivatives (3.83%), pyridines and derivatives (2.59%), prenol lipids (2.59%), and flavonoids (2.10%) (Figure 3D).

The different metabolites were assigned to the KEGG pathway record to find the relevant pathways of metabolism. The KEGG pathway classification revealed that a total of 666 DEMs were mapped on different pathways. The amino acid metabolism pathway grasped the largest number of compounds, succeeded by the production of other secondary metabolites, xenobiotics biodegradation, chemical structure alteration maps, carbohydrate metabolism, and membrane transport (Figure 3E).

### 3.4. Enrichment Analysis of KEGG Pathway and Differential Abundance Score

The distinct metabolites were tracked on the KEGG repository, and the KEGG pathway enrichment assessment was performed to examine the biological functions of molecules of various treatments. The differential abundance score (DAS) was utilized to assess the rise or reduction of metabolites in certain metabolism pathways. The enrichment ratio for the most promising 20 KEGG pathways was examined.

**Figure 4:**
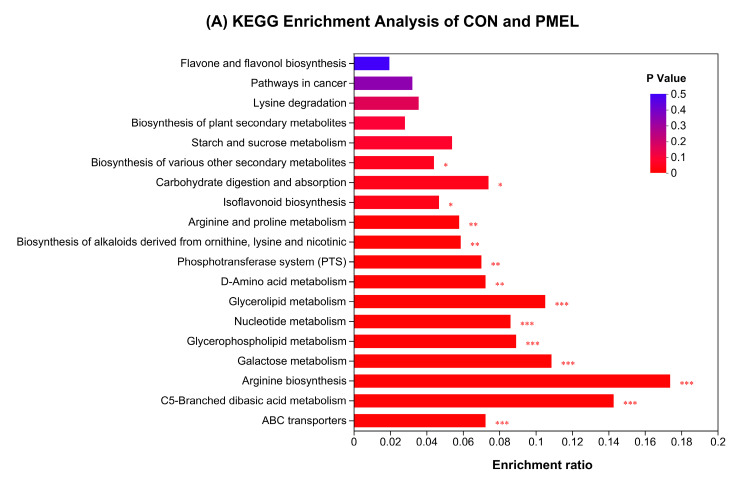

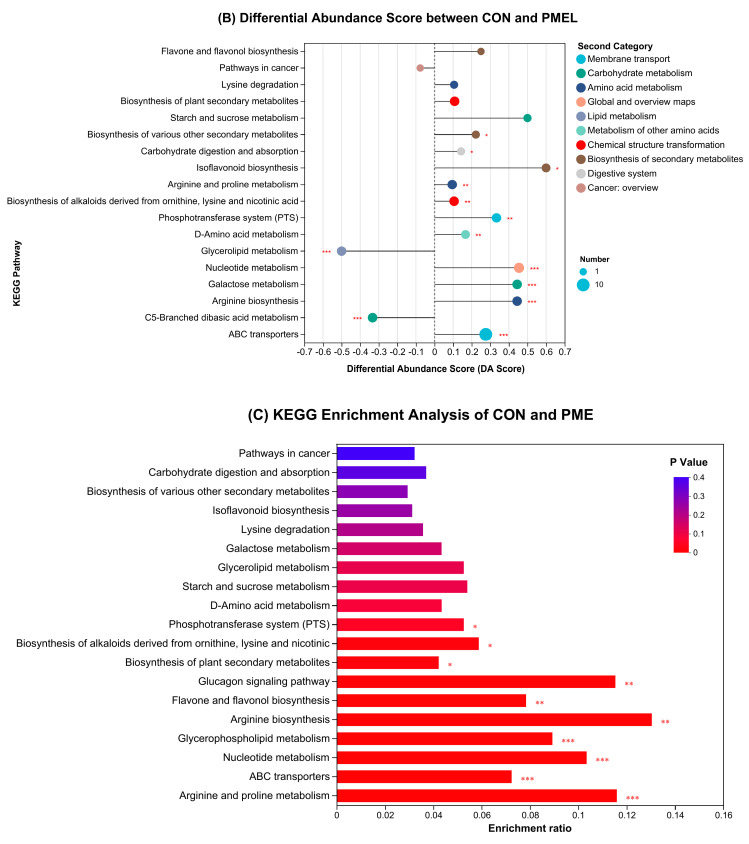

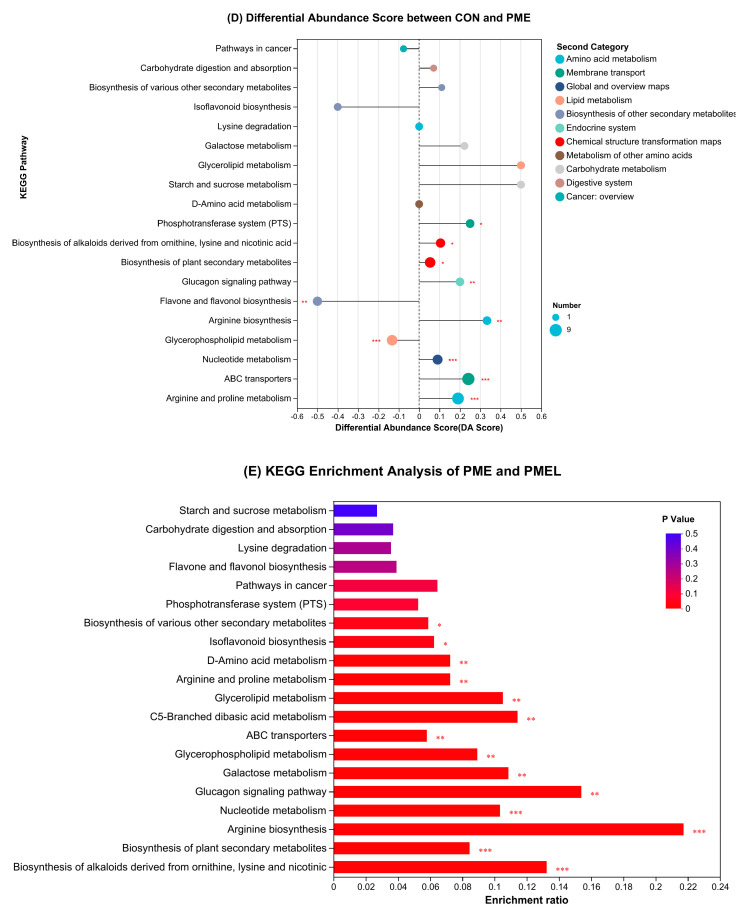

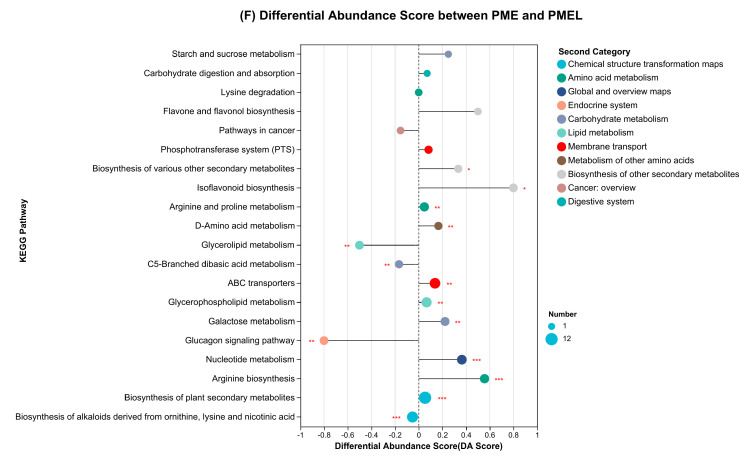
Enrichment analysis of KEGG pathway and Differential Abundance Score: (**A**) KEGG enrichment analysis of CON and PMEL; (**B**) Differential Abundance Score between CON and PMEL; (**C**) KEGG enrichment analysis of CON and PME; (**D**) Differential Abundance Score between CON and PME; (**E**) KEGG enrichment analysis of PME and PMEL, and (**F**) Differential Abundance Score between PME and PMEL. *** *p* < 0.001; ** *p* < 0.01; * *p* < 0.05.

In CON vs. PMEL, the highest enrichment ratio (*p* < 0.001) was noted in PME for arginine biosynthesis, followed by C5-Bracnhed dibasic acid metabolism, galactose metabolism, glycerolipid metabolism, and glycerophospholipid metabolism. Apart from this, pathways related to the biosynthesis of isoflavonoid and various other secondary metabolites were also significantly enriched pathways (*p* < 0.05); however, flavone and flavonol biosynthesis pathways tended to be enriched pathways in PMEL (Figure 4A). The DAS of CON vs. PMEL revealed that the glycerolipid metabolism pathway was significantly downregulated (*p* < 0.001), whereas the pathways having significantly upregulated (*p* < 0.001) compounds were concerned with arginine biosynthesis and galactose metabolism. The isoflavonoid biosynthesis and biosynthesis of other various secondary metabolites were also significantly upregulated pathways (*p* < 0.05); however, flavone and flavonol biosynthesis pathways tended to be upregulated pathways in PMEL (Figure 4B).

The KEGG enrichment assessment of CON vs. PME showed that metabolic pathways linked to the ABC transporters, nucleotide metabolism, metabolism of arginine and proline, and glycerophospholipid metabolism had the highest augmented ratio in PME (*p* < 0.001). Similarly, the flavone and flavonol biosynthesis and arginine biogenesis pathways were also substantially improved pathways in PME (*p* < 0.01) (Figure 4C). The DAS evaluation of CON versus PME revealed that the chemical pathways showing increasing trends (*p* < 0.001) were related to ABC transporters, nucleotide metabolism, and arginine and proline metabolism in PME. The pathway indicating the downregulation (*p* < 0.05) of metabolites was relevant to flavone and flavonol biosynthesis; however, the isoflavonoid biosynthesis pathway tended to be downregulated in PME (Figure 4D).

The KEGG enrichment examination of PME versus PMEL discovered that metabolic processes linked to the biosynthesis of alkaloids, arginine biosynthesis, and biosynthesis of plant secondary metabolites possessed an enrichment ratio that was noticeably higher in PMEL (*p* < 0.001). The pathways related to the biosynthesis of isoflavonoids and other various secondary metabolites were also substantially augmented (*p* < 0.05). However, flavone and flavonol biosynthesis pathways tended to be enriched pathways in PMEL (Figure 4E). The DAS of PME vs. PMEL revealed that the pathways having significantly upregulated (*p* < 0.001) biomolecules were linked to arginine biosynthesis and nucleotide metabolism. The isoflavonoid biosynthesis and biosynthesis of other various secondary metabolites were also significantly upregulated pathways (*p* < 0.05); however, flavone and flavonol biosynthesis pathways tended to be upregulated pathways in PMEL (Figure 4F). These findings suggested that the fermentation with *Lactobacillus* yielded an increased enrichment ratio and upregulation of isoflavonoid biosynthesis and biosynthesis of other various secondary metabolites pathways in PMEL, which, in turn, pre-eminent the antioxidant capability of PMEL.

### 3.5. Metabolites Possessing Antioxidant Potential and Their VIP Assessment, Total Flavonoids and Polyphenols Content, and Antioxidant Capability

**Figure 5:**
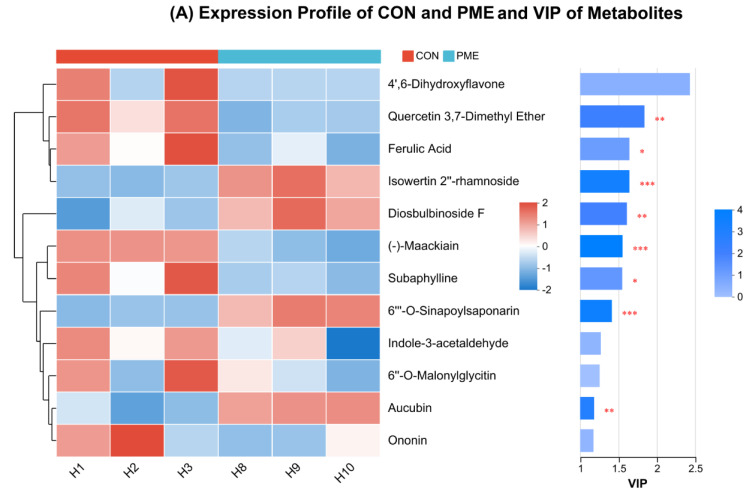

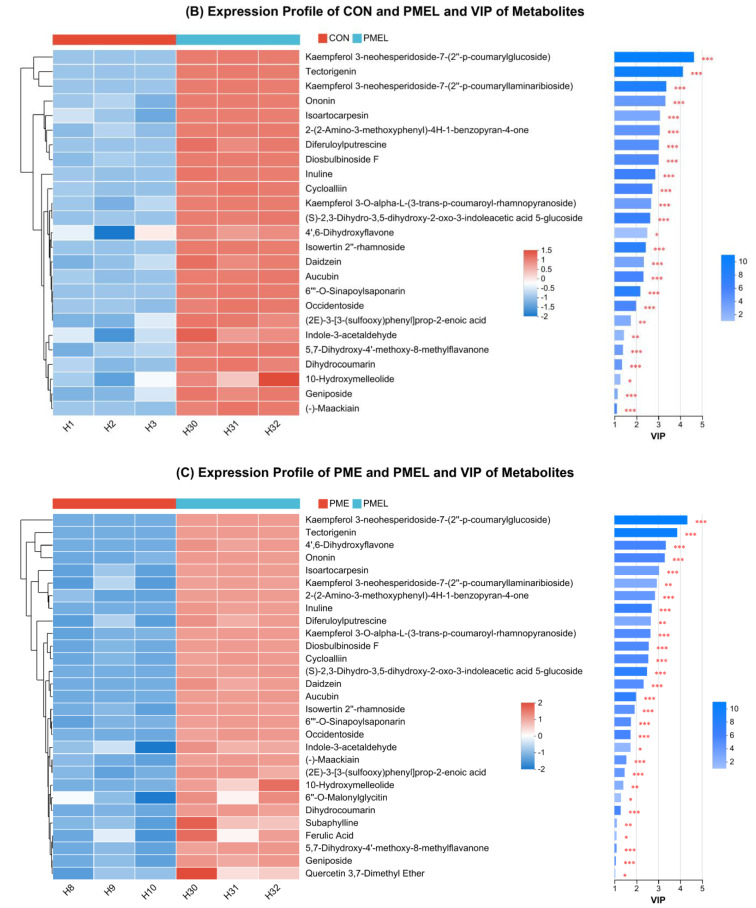

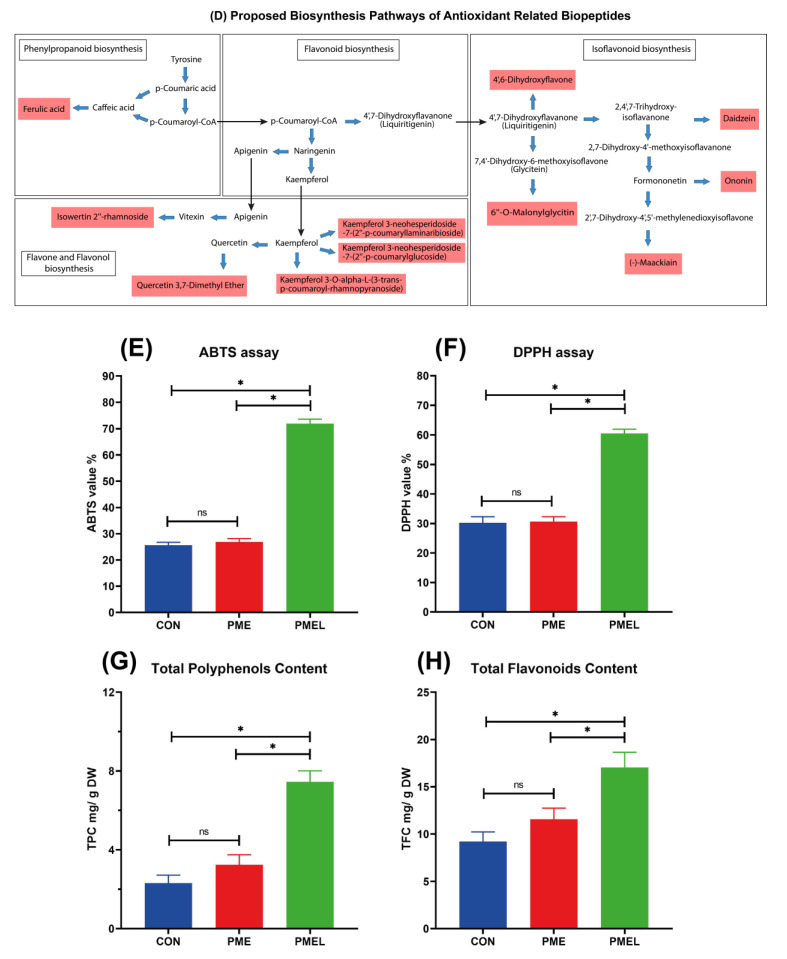
Heatmap Analysis and VIP of metabolites related to antioxidant activity, scavenging values, total flavonoids, and total polyphenols content. (**A**) Expression profile of CON and PME and VIP of metabolites. The significantly upregulated metabolites in PME were diosbulbinoside, aucubin, isowertin 2″-rhamnoside, and 6‴-O-sinapoylsaponarin; (**B**) Expression profile of CON and PMEL and VIP of metabolites. All the mentioned metabolites in PMEL were significantly upregulated; (**C**) Expression profile of PME and PMEL and VIP of metabolites. All the mentioned metabolites in PMEL were significantly upregulated; (**D**) Proposed biosynthesis pathway of antioxidant-related peptides. Mainly, 4 biosynthesis pathways were identified, namely phenylpropanoid, isoflavonoid, flavonoid, and flavone and flavonol biosynthesis pathways. The p-coumaroyl-CoA generation is crucial because its successors are liquiritigenin, kaempferol, and apigenin. The kaempferol is then converted to kaempferol derivatives and quercetin. The quercetin is finally converted to quercetin 3,7-dimethyl ether. The liquiritigenin conversion to its successors like daidzein, ononin, and maackiain in the isoflavonoid biosynthesis pathway is very crucial because it may regulate the antioxidant status of fermented PMEL; (**E**) ABTS analysis to check the scavenging value; (**F**) DPPH analysis to check the scavenging value; (**G**) Total polyphenols content and (**H**) total flavonoids content. *** *p* < 0.001; ** *p* < 0.01; * *p* < 0.05; ns, non-significant.

To determine the substantial distinction in metabolites between the two groups, the compounds possessing antioxidant-related properties (flavonoids, isoflavonoids, lignan glycosides, cinnamic acids, dihydrocoumarins, indoles, and prenol lipids) were scrutinized, administered to the OPLS-DA approach, and the VIP score was assessed. The expression profile of CON and PME demonstrated that 12 metabolites were discovered, with diosbulbinoside, aucubin, isowertin 2″-rhamnoside, and 6‴-O-sinapoylsaponarin being considerably more prevalent (*p* < 0.01) in PME (Figure 5A).

A total of 25 metabolites were detected in the expression profile of CON and PMEL. The hydroxymelleolide, 4′,6-Dihydroxyflavone, and indole-3-acetaldehyde were notably increased (*p* < 0.01) compounds in the PMEL group whereas the extremely noteworthy (*p* < 0.001) metabolites in PMEL were methylflavanone, kaempferol derivatives, daidzein, geniposide, isoartocarpesin, dihydrocoumarin, occidentoside, maackiain, cycloalliin, aucubin, inuline, isowertin 2″-rhamnoside, tectorigenin, ononin, diferuloylputrescine, diosbulbinoside, indoleacetic acid 5-glucoside, and sinapoylsaponarin (Figure 5B).

The expression assessment of PME and PMEL confirmed that 29 metabolites were significantly different, and the notably highest (*p* < 0.001) metabolites were occidentoside, methylflavanone, kaempferol derivatives, daidzein, isoartocarpesin, dihydrocoumarin, maackiain, cycloalliin, aucubin, inuline, isowertin 2″-rhamnoside, tectorigenin, ononin, and indoleacetic acid 5-glucoside in PMEL. Similarly, ominously prominent (*p* < 0.05) metabolites detected in PMEL were malonylglycitin, ferulic acid, subaphylline, quercetin 3,7-dimethyl ether, and indole-3-acetaldehyde (Figure 5C). These findings verified that *Lactobacillus* fermentation considerably advanced the flavonoids, isoflavonoids, lignan glycosides, cinnamic acids, dihydrocoumarins, indoles, and prenol lipids in PMEL, which triggered a significant upsurge in the entire antioxidant ability of fermented PKM.

The anticipated biosynthesis pathways of antioxidant-related bioactive peptides are discussed in Figure 5D. Mainly, four biosynthesis pathways were identified, namely phenylpropanoid, isoflavonoid, flavonoid, and flavone and flavonol biosynthesis pathways. The p-coumaric acid is generated from tyrosine in the phenylpropanoid biosynthesis pathway and is converted to p-coumaroyl-CoA and caffeic acid. The caffeic acid is then converted to ferulic acid. After entering the flavonoid biosynthesis pathway, the p-coumaroyl-CoA is transformed into liquiritigenin and naringenin. The naringenin is converted to kaempferol and apigenin, and later, both enter the flavone and flavonol biosynthesis pathway. The kaempferol is then converted to kaempferol derivatives and quercetin. The quercetin is finally converted to quercetin 3,7-dimethyl ether. The liquiritigenin from the flavonoid biosynthesis pathway flows into the isoflavonoid biosynthesis pathway, where it is converted to 4′,6-dihydroxyflavone, 2,4′,7-trihydroxy-isoflavanone, and glycitein. The glycitein is then converted to 6″-O-malonylglycitin. The 2,4′,7-trihydroxy-isoflavanone is converted to daidzein and 2,7-dihydroxy-4′-methoxyisoflavanone. The latter one is then converted to formononetin, which is then converted to ononin and 2′,7-dihydroxy-4′,5′-methylenedioxyisoflavone. The latter one is finally converted to maackiain (Figure 5D).

The overall antioxidant potential of cultured PKM was assessed by employing a DPPH and ABTS test. The DPPH and ABTS scavenging efficiencies of CON and PME appeared equivalent. Interestingly, the DPPH and ABTS scavenging rates of PMEL were substantially greater (*p* < 0.05) as opposed to CON and PME (Figure 5E,F). The overall flavonoid and polyphenol levels of PMEL were considerably stronger (*p* < 0.05) compared to that of the remaining groups (Figure 5G,H). There existed no difference in the overall flavonoid or polyphenol values of CON and PME. The increased levels of polyphenols and flavonoids in the PMEL probably assisted in its elevated overall antioxidant efficacy.

### 3.6. Metagenome of Bacterial Microbial Community

**Figure 6:**
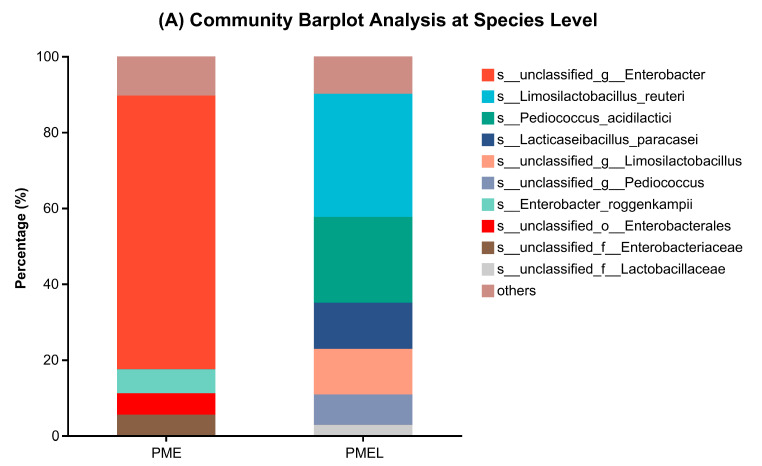

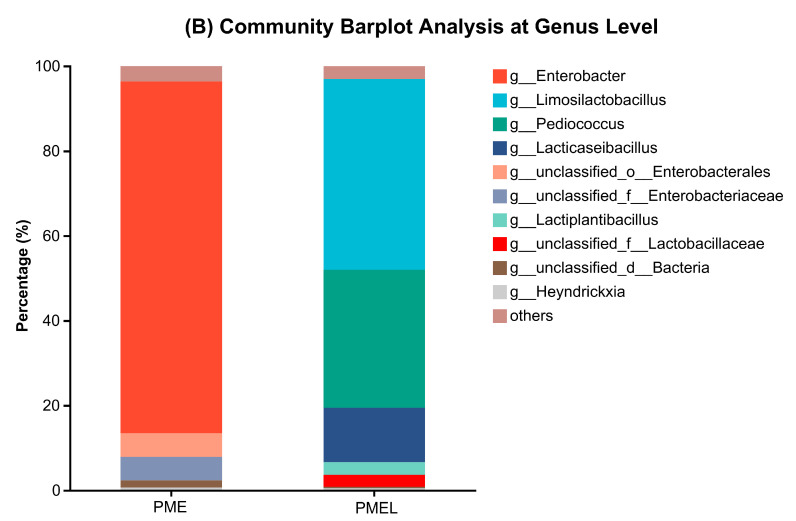

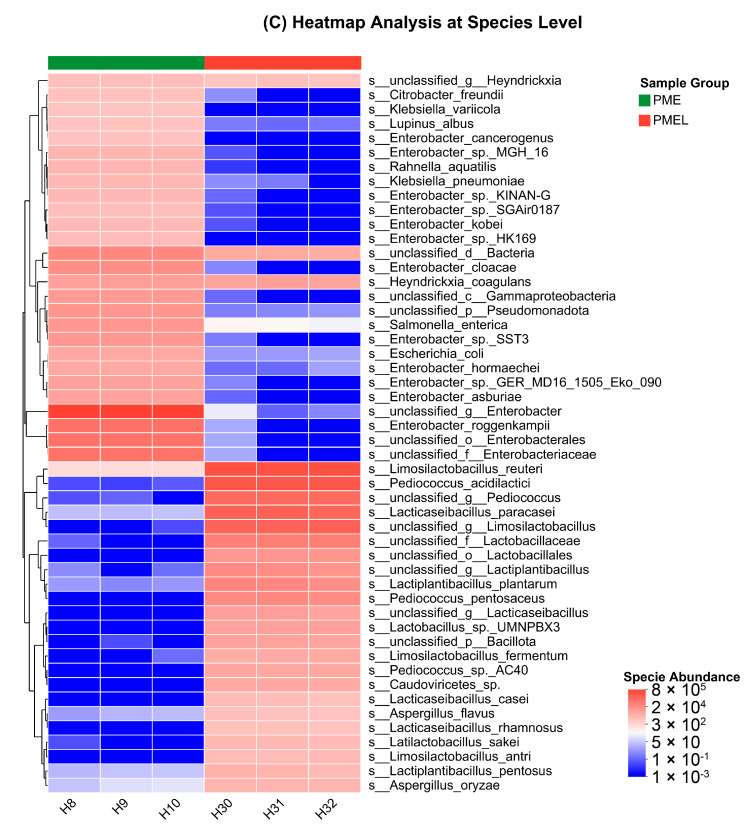

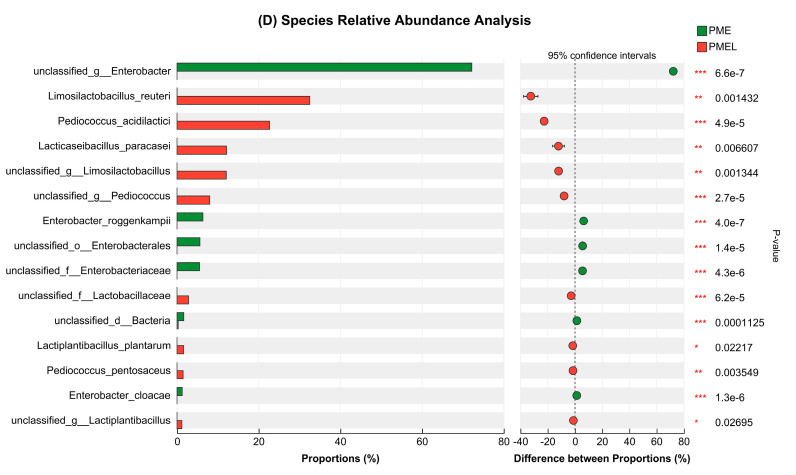
Metagenomic analysis of bacterial microbial community. (**A**) Community barplot analysis of PME and PMEL at the species level; (**B**) Community barplot analysis of PME and PMEL at genus level; (**C**) Species heatmap analysis of PME and PMEL, and (**D**) species relative abundance analysis of PME and PMEL. The PME mainly possessed *s__unclassified_g__Enterobacter* (72.16%) species, which might be responsible for CAZy in PME. The PMEL consisted of *Limosilactobacillus reuteri* (32.49%) and *Pediococcus acidilactici* (22.66%), which may have contributed to its CAZy and antioxidant production. *** *p* < 0.001; ** *p* < 0.01; * *p* < 0.05.

To assess the bacterial microbial community of PME and PMEL, 16sRNA sequencing was performed. Generally, the most dominant microbial community in PME was related to the order *Enterobacterales*, whereas in PMEL, it was related to the order *Lactobacillales*. At the species level, the PME mainly possessed *s__unclassified_g__Enterobacter* (72.16%) and *Enterobacter roggenkampii* (6.34%), whereas PMEL consisted of *Limosilactobacillus reuteri* (32.49%), *Pediococcus acidilactici* (22.66%), and *Lacticaseibacillus paracasei* (12.14%) (Figure 6A). At the genus level, the PME mainly comprised the genus *Enterobacter* (82.95%) and the unclassified genus having the family *Enterobacteriaceae* (5.53%), whereas the PMEL mainly included the genus *Limosilactobacillus* (44.99%), *Pediococcus* (32.54%), *Lacticaseibacillus* (12.76%), and *Lactiplantibacillus* (3.03%) (Figure 6B).

The heatmap analysis was performed to determine the species abundance and composition in PME and PMEL. The dominant species in PME were *Enterobacter roggenkampii*, *Enterobacter asburiae*, *Enterobacter hormaechei*, and *Escherichia coli*, whereas, in PMEL, the dominant species were *Limosilactobacillus reuteri*, *Pediococcus acidilactici*, *Lacticaseibacillus paracasei*, and *Lactiplantibacillus plantarum* (Figure 6C).

The species relative abundance analysis revealed that *Limosilactobacillus reuteri*, *Pediococcus acidilactici*, *Lacticaseibacillus paracasei*, *Pediococcus pentosaceus*, and *Lactiplantibacillus plantarum* had significantly higher (*p* < 0.05) proportion in PMEL as compared to PME, whereas in PME, *s__unclassified_g__Enterobacter*, *Enterobacter roggenkampii,* and *Enterobacter cloacae* showed significantly higher (*p* < 0.001) proportion as compared to PMEL (Figure 6D). These results demonstrated that PMEL harbored more *Lactobacillus* species and had a diverse bacterial community.

### 3.7. Carbohydrates Active Enzymes (CAZy) Analysis

**Figure 7:**
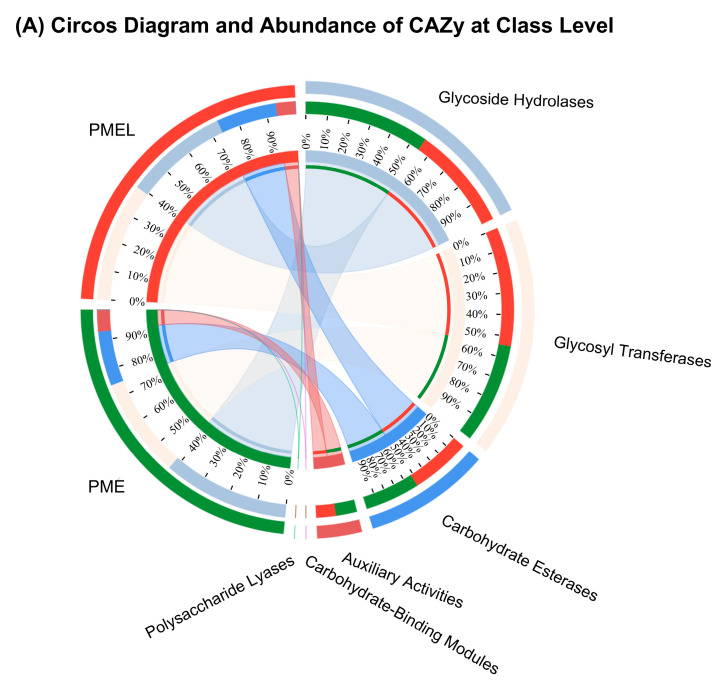

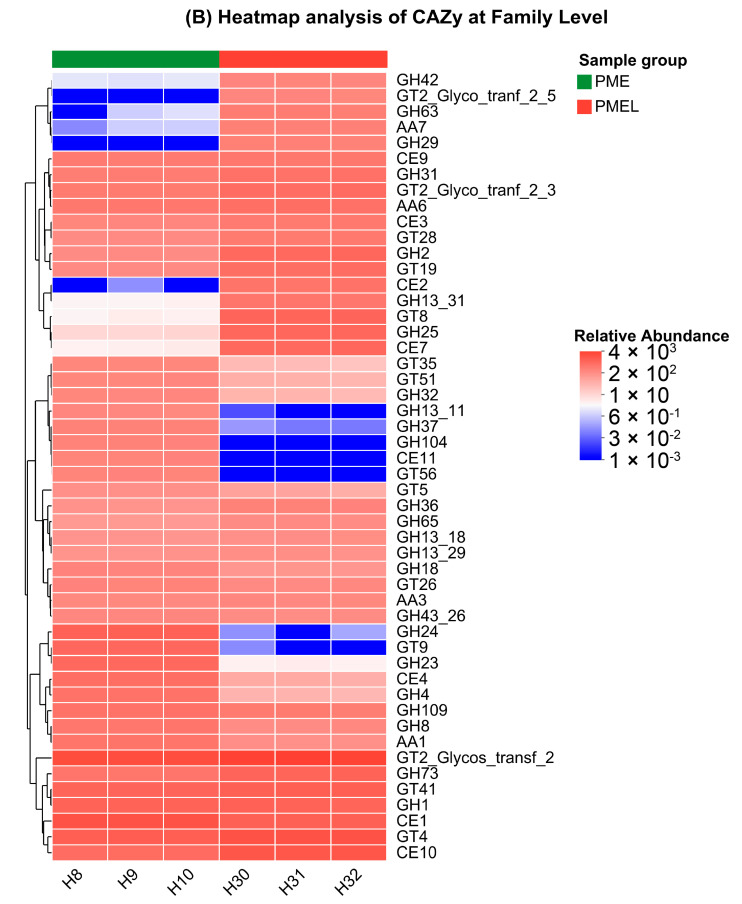

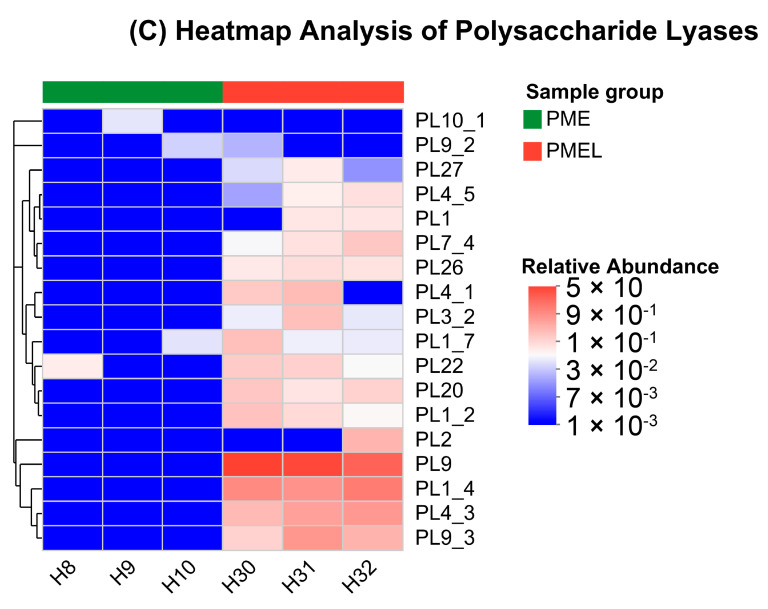

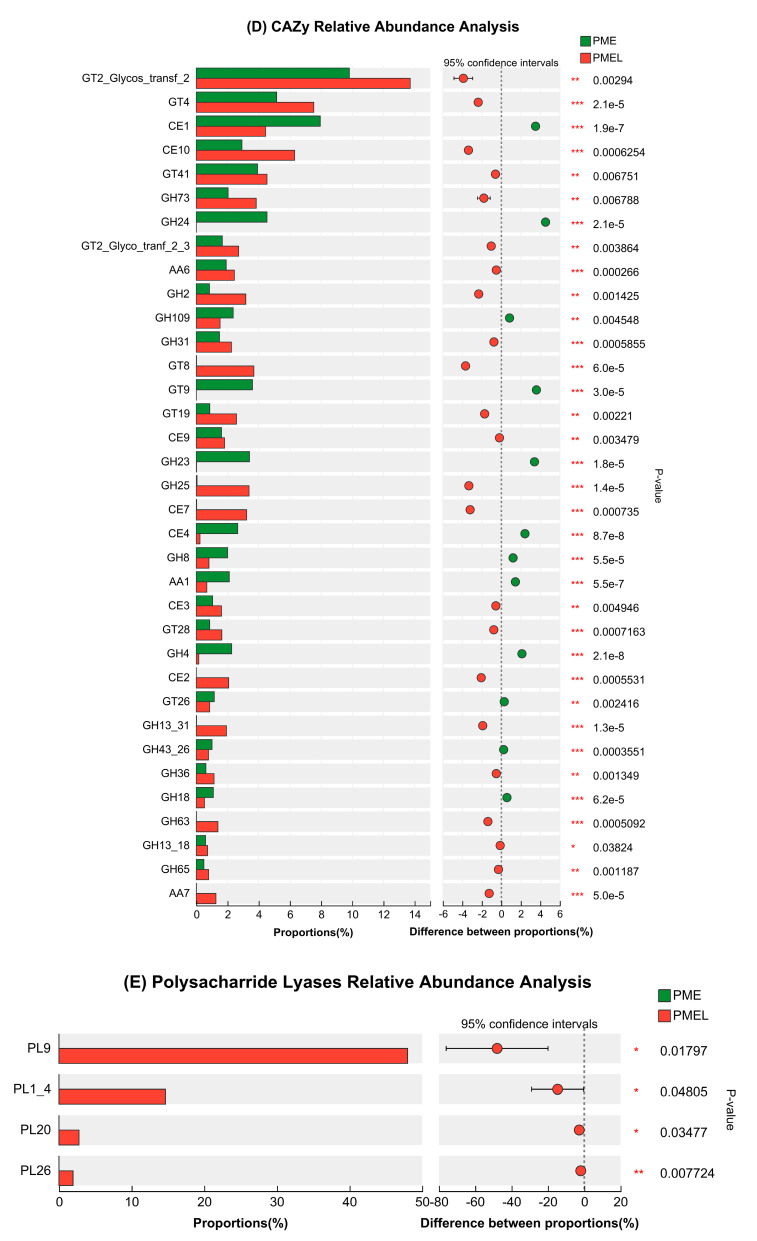

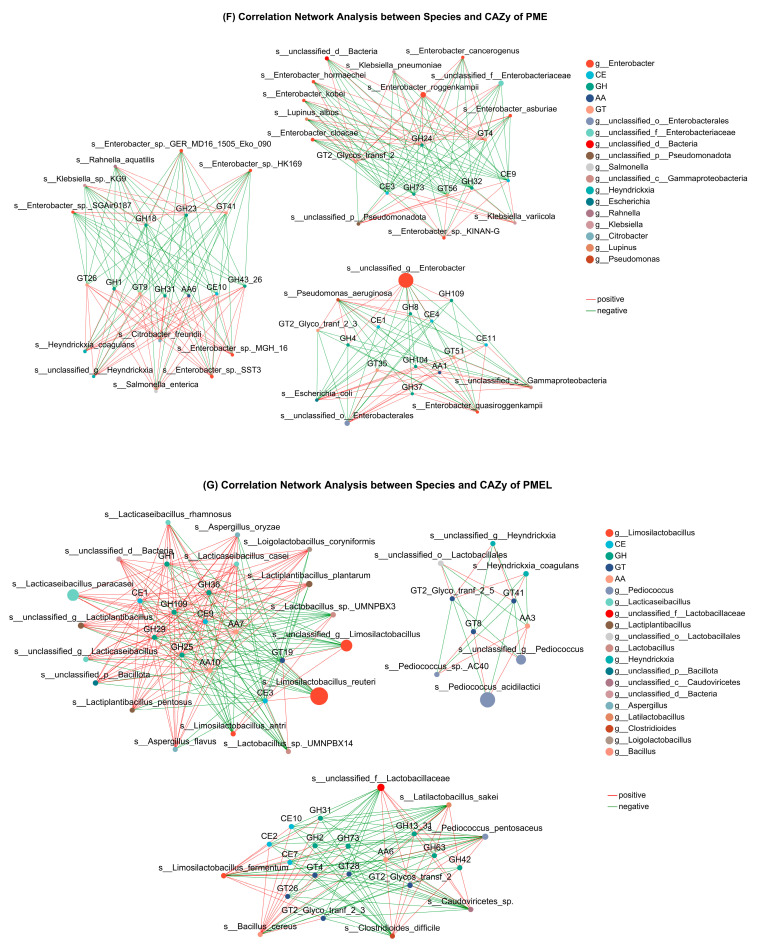
CAZy analysis. (**A**) Circos diagram and abundance of CAZy of PME and PMEL, (**B**) Heatmap analysis of CAZy of PME and PMEL at the family level, (**C**) Heatmap analysis of polysaccharide lyases of PME and PMEL, (**D**) CAZy relative abundance analysis between PME and PMEL, (**E**) Polysaccharide lyases relative abundance analysis between PME and PMEL, (**F**) Correlation network analysis among species and CAZy of PME, and (**G**) Correlation network analysis among species and CAZy of PMEL. The PMEL had significantly higher (*p* < 0.01) abundance levels of GT4, GT2_Glycos_transf_2, GH73, GT41, CE10, GH2, AA6, GT8, GH31, and GT2_Glyco_tranf_2_3, which possess the cellulose synthase, xylanase, chitin synthase, endoglucanase, and beta-galactosidase-like activity. The PMEL also possessed a significantly higher (*p* < 0.05) relative abundance of PL26, PL20, PL1_4, and PL9, which may be responsible for the degradation of the cell wall, specifically pectin complex polysaccharides. *** *p* < 0.001; ** *p* < 0.01; * *p* < 0.05.

The circos diagram illustrated the CAZy in the PME and PMEL groups. The PME had six classes of enzymes, namely glycoside hydrolases (GH) (42%), glycosyl transferases (GT) (33%), carbohydrate esterases (CE) (18%), auxiliary activities (AA) (7%), carbohydrate-binding modules (0.005%), and polysaccharide lyases (PL) (0.0001%). The PMEL also had six classes of enzymes, namely glycoside hydrolases (34%), glycosyl transferases (39%), carbohydrate esterases (20%), auxiliary activities (6%), carbohydrate-binding modules (0.008%), and polysaccharide lyases (0.03%) (Figure 7A).

The heatmap analysis of CAZy at the family level revealed that PME had a higher relative abundance of GH13_11, GH37, GH104, CE11, GT56, GT9, and GH24 families, whereas PMEL comprised a higher relative abundance of GH42, GT2_Glyco_tranf_2_5, GH63, AA7, GH29, CE2, GH13_31, GT8, GH25, and CE7 families (Figure 7B). The relative abundance of PL families in PME and PMEL varies greatly. The heatmap analysis showed that PMEL possessed a higher relative abundance of different PL families (PL9, PL1_4, PL4_3, PL9_3, PL22, PL20, and PL1_2) as compared to the PME (Figure 7C).

The relative abundance analysis of CAZy showed that PMEL had significantly higher (*p* < 0.01) abundance levels of GT4, GT2_Glycos_transf_2, GH73, GT41, CE10, GH2, AA6, GT8, GH31, and GT2_Glyco_tranf_2_3 as compared to PME (Figure 7D). Similarly, PMEL possessed a significantly higher (*p* < 0.05) relative abundance of PL26, PL20, PL1_4, and PL9 as compared to PME (Figure 7E).

The correlation network assessment was accomplished to comprehend the association among microbes and enzymes. The correlation network analysis between PME and CAZy exposed that *Pseudomonas aeruginosa*, *Escherichia coli*, *Enterobacter quasiroggenkampii*, *Salmonella enterica*, and *Heyndrickxia coagulans* were mainly responsible for CAZy occurrence in PME (Figure 7F). However, the correlation network analysis between PMEL and CAZy revealed that *Lactiplantibacillus plantarum*, *Limosilactobacillus reuteri*, *Latilactobacillus sakei*, *Pediococcus pentosaceus*, *Bacillus cereus*, *Limosilactobacillus fermentum*, *Aspergillus oryzae*, *Aspergillus flavus*, and *Lacticaseibacillus rhamnosus* were mainly accounted for the CAZy development in PMEL (Figure 7G). The above results demonstrated that *Lactobacillus* fermentation led to a diversified microbial community and was responsible for developing various CAZy in PMEL, ultimately boosting its nutritional and antioxidant value.

### 3.8. Species and Metabolites Correlation Analysis and WGCNA

**Figure 8:**
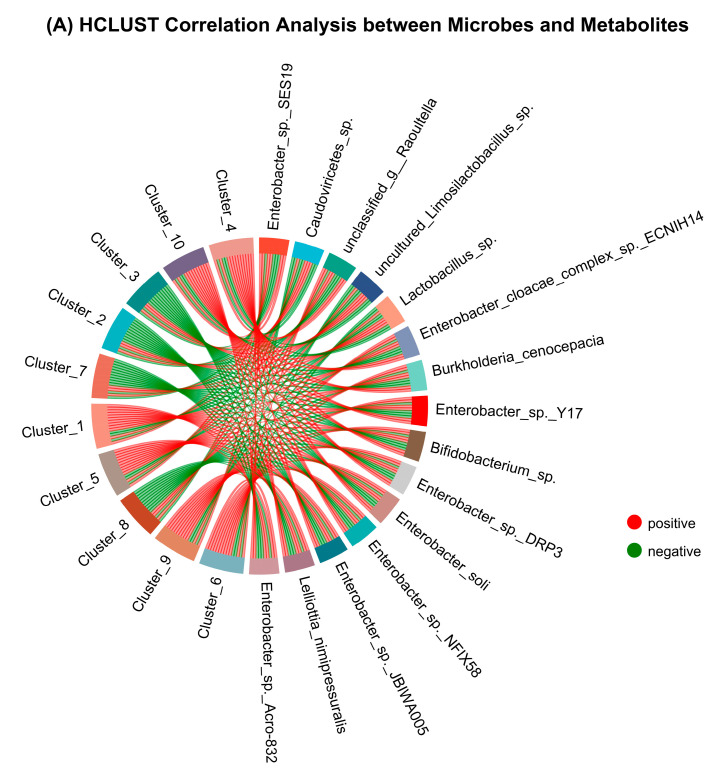

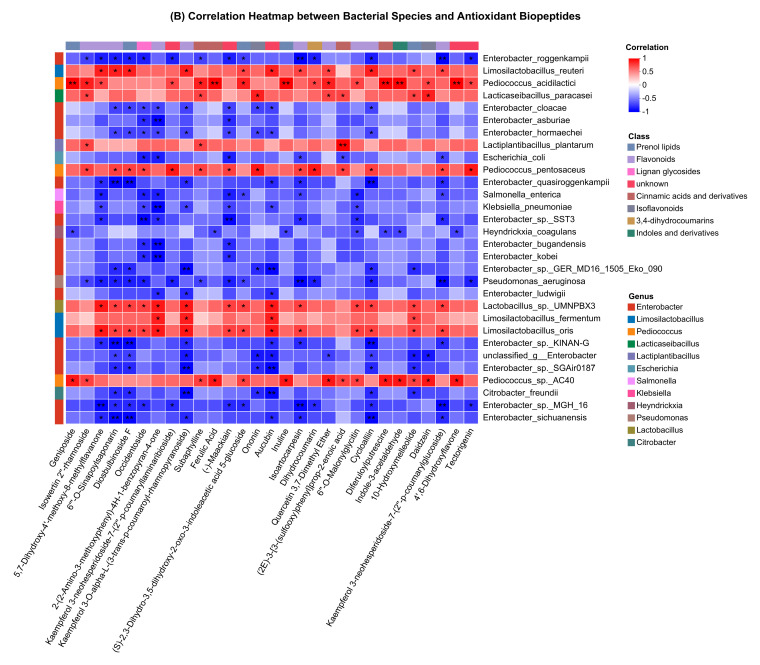

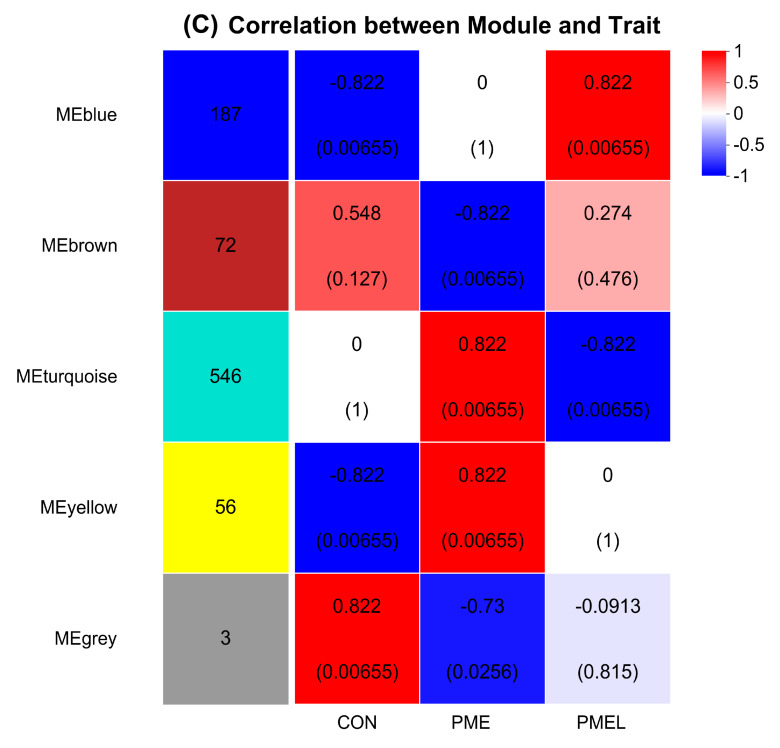

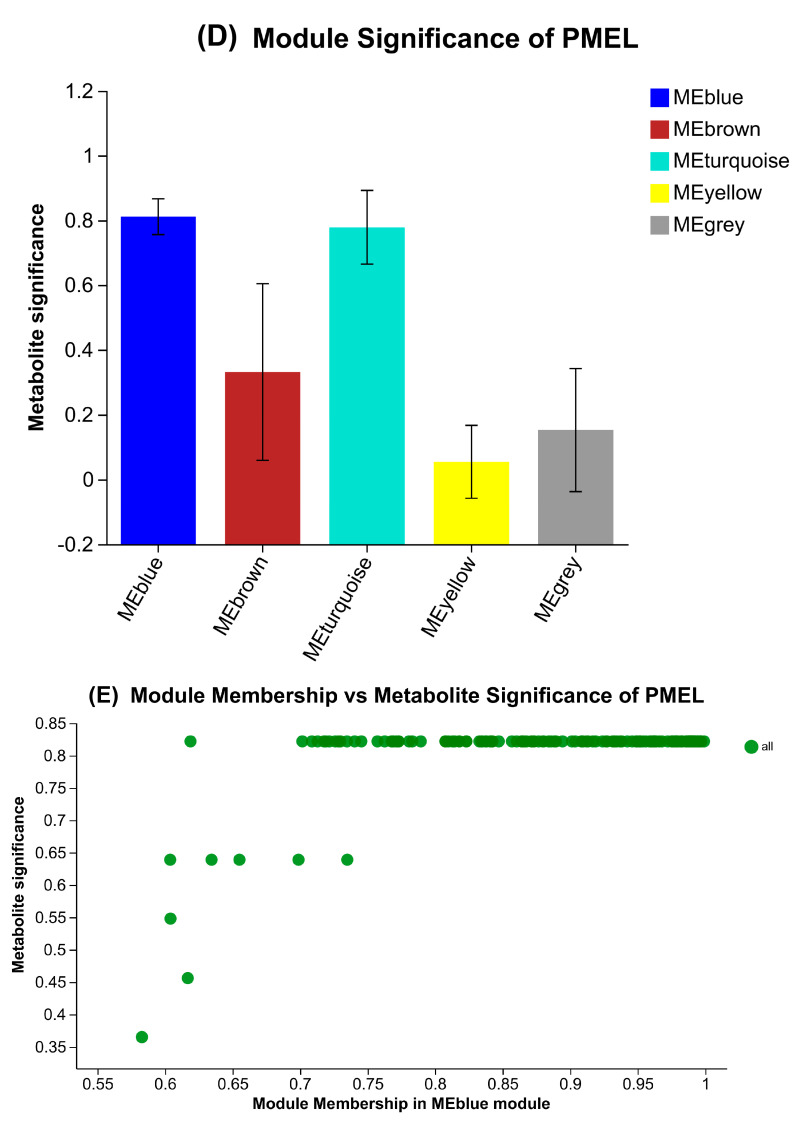

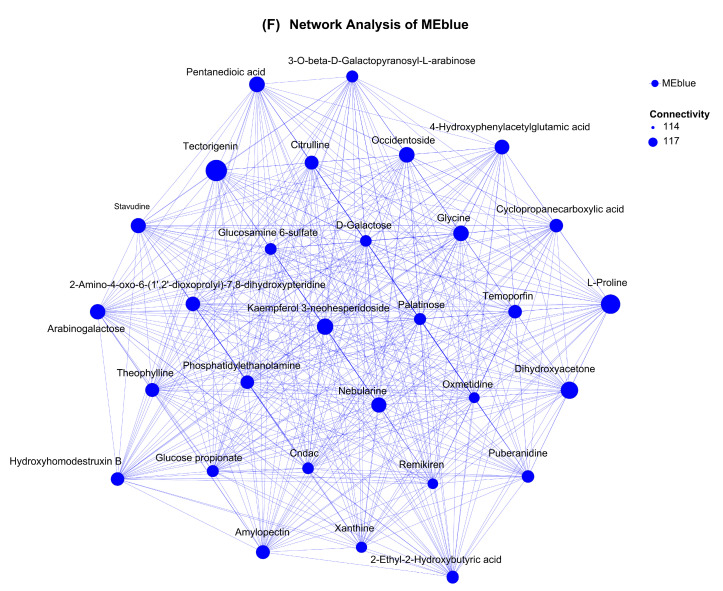
Species and Metabolites Correlation Analysis and WGCNA: (**A**) HCLUST correlation analysis between microbes and metabolites; (**B**) Correlation heatmap between bacterial species and antioxidant biopeptides; (**C**) Correlation between module and trait; (**D**) Module significance of PMEL; (**E**) Module membership vs. metabolite significance of PMEL; and (**F**) Network analysis of MEblue. ** *p* < 0.01; * *p* < 0.05.

Significant clusters were identified by the HCLUST analysis, suggesting strong relationships between certain microbial populations and metabolites. These clusters indicate possible functional connections or interactions among the components of the microbial community. The analysis revealed important compounds that are significantly associated with specific microbial groups, therefore emphasizing potential metabolic pathways. Clusters 2, 3, 7, and 8 were positively correlated with *Lactobacillus species.* Whereas clusters 1, 5, 6, 9, and 10 were positively correlated with *Enterobacter species* (Figure 8A).

The correlation heatmap analysis between bacterial species and antioxidant peptides revealed that most of the *Lactobacillus* and *Pediococcus species* were positively correlated with antioxidant peptides, whereas *Enterobacter species* were negatively correlated with antioxidant peptides. The *Enterobacter roggenkampii*, *Enterobacter cloacae*, *Enterobacter asburiae*, and *Enterobacter hormaechei* were considerably adversely (*p* < 0.05) correlated with antioxidant peptides, and these species have been reported in PME. The *Limosilactobacillus reuteri*, *Pediococcus acidilactici*, *Lacticaseibacillus paracasei*, *Lactiplantibacillus plantarum*, *Pediococcus pentosaceus*, *Limosilactobacillus fermentum*, and *Limosilactobacillus oris* were considerably positively (*p* < 0.05) interrelated with antioxidants peptides and these bacterial species have been found in PMEL (Figure 8B). The above findings implied that *Lactobacillus* fermentation enhances the *Lactobacillus* and *Pediococcus species* in PMEL, which were positively correlated with antioxidant peptides and hence increased its antioxidant potential.

In the context of metabolome data, Weighted Gene Co-expression Network Analysis (WGCNA) was employed to detect clusters (modules) of strongly correlated metabolites, evaluate the association between these modules and exterior traits, and identify significant metabolites that could potentially function as biomarkers of antioxidant activity. The sample clustering dendrogram, metabolites dendrogram, correlation between modules, and module clustering dendrogram can be found in Supplementary Material (Figure S1). The correlation between module and trait revealed that MEblue was significantly (*p* < 0.05) positively correlated, and MEturquoise was significantly (*p* < 0.05) negatively correlated with PMEL (*Lactobacillus* fermentation), whereas MEturquoise and MEyellow had a significant (*p* < 0.05) positive association with PME (Figure 8C). The module significance of PMEL revealed that MEblue and MEturquoise had more significance as compared to other modules (Figure 8D). To understand the MEblue significance, we performed (MM-GS analysis) module membership vs. gene/metabolite significance analysis, which revealed that most of the metabolites depicted higher MM and GS and were responsible for variation in PMEL (Figure 8E). The network analysis of MEblue revealed the hub metabolites, which included different antioxidant peptides like tectorigenin and kaempferol (Figure 8F).

## 4. Discussion

Integrating metabolomics with metagenomics has been demonstrated to be an effective strategy for deciphering complicated biological processes along with finding novel biomarkers with significant applications across numerous fields. The current research used an integrated approach to investigate the antioxidant potential of fermented palm kernel meal. These products exhibit remarkable antioxidant activity and demonstrate the potential to serve as a functional food additive and bioactive supplement in animals’ diets to mitigate toxicity owing to their exceptional nutritional composition and bioactive substances [46,47].

*Lactobacillus* is a strain of Gram-positive bacteria capable of flourishing in both anaerobic and aerobic conditions. Through carbohydrate fermentation, it is capable of generating two distinct isomers of lactic acid [48]. Due to its exceptional probiotic, antioxidant, and immune-enhancing properties, it has been widely used as a probiotic [49,50]. The current research project utilized integrative metabolomic and metagenomic approaches to acquire a deep understanding of the distinguishing aspects of the metabolome and metagenome, along with the antioxidant-related substances in PKM fermented with *L. plantarum* and *L. reuteri*.

### 4.1. Fermented Palm Kernel Meal, Metabolomic, and Metagenomic Profile

The metabolomics assessment presented a thorough understanding of the biochemical features of fermented PKM. Employing high-throughput approaches, i.e., high-performance liquid chromatography-mass spectrometry (HPLC-MS), we discovered a wide range of metabolites comprising phenolic substances, isoflavonoids, flavonoids, lignan glycosides, cinnamic acids, prenol lipids, and indoles derivatives. These biomolecules are well-recognized for their antioxidant capabilities and perform significant responsibilities in eliminating free radicals, decreasing oxidative damage, and imparting positive health effects [51,52].

The heatmap suggested a substantial upregulation of flavonoids and isoflavonoids in the PMEL group. The prominent flavonoids such as dihydroxyflavone, isowertin, methylflavanone, saponarin, benzopyran, quercetin, isoartocarpesin, cycoalliin, and kaempferol derivatives were substantially upregulated in PMEL. The flavonoids have been extensively reported for their involvement in decreasing the risk of chronic illnesses via their antioxidant and anti-inflammatory activities [53,54,55]. Dihydroxyflavone (flavones) illustrates antioxidant capabilities and neuroprotective benefits towards glutamate-induced toxic effects [56]. The antioxidant action of methylflavanone (O-methylated flavonoids) could include processes such as effective quenching of free radicals and stimulation of cellular detoxifying enzymes [57]. The benzopyrans (O-methylated flavonoids) possess antioxidative properties, which could be linked to their possible medicinal implementations in oxidative stress-related disorders [58]. The isoartocarpesin is a flavone that functions as a powerful antioxidant by regulating surplus reactive oxygen species (ROS) generation [59]. The kaempferol derivatives products are flavonoid glycosides that exhibit diversified biological properties comprising antioxidant, anticancer, and anti-inflammatory capabilities [60]. This demonstrated that *Lactobacillus* fermentation boosted flavonoid concentration and, consequently, the antioxidant properties of PMEL.

The isoflavonoids, including tectorigenin, maackiain, daidzein, malonylglycitin, and ononin, exhibited a considerable increase in PMEL. These isoflavonoids are renowned for their antioxidant capabilities, which help to lower the stress caused by oxidative damage [61,62,63]. Tectorigenin is an organic chemical categorized as an isoflavone that has many biological actions, which include anti-inflammatory, antioxidant, cancer-fighting, and estrogen-like properties [64]. Daidzein is an isoflavone that performs its antioxidant action by preventing the peroxidation of lipids in the membranes of cells, preventing renal impairment [65]. Ononin is an organic isoflavone glycoside typically found in many plants and displays antioxidant capabilities, which are connected to minimizing ROS formation and the amount of inflammatory mediators [66]. The elevated amounts of these isoflavonoids revealed *Lactobacillus* fermentation boosted the antioxidant ability of PMEL.

The impact of the *Lactobacillus* fermentation process on the isoflavonoid biosynthesis pathway in PKM has been thoroughly researched. Our work found that the isoflavonoids production pathway exhibited significant enrichment ratios in PMEL, which led to improved antioxidant capability. Similar findings have been published earlier. An investigation by Merenkova et al. [67] highlighted the synthesis of isoflavone aglycones in fermented grains, suggesting the prospect for enhanced isoflavonoid yield via fermentation techniques. Likewise, Verni et al. [68] examined the modification of the formation mechanism of flavonoids through fermentation, demonstrating an increase in isoflavone percentage post-fermentation. Yang et al. [69] further corroborated these findings, revealing an enormous improvement in isoflavone levels in polyphenol-rich foodstuffs after the process of fermentation. In other research, Gao et al. [70] evaluated the flavonoid production during alfalfa silage microbial fermentation and reported a surge in the antioxidant capacity post-fermentation. These findings collectively indicate the intricate connection between fermentation, isoflavonoid synthesis, and antioxidant properties, explaining the potential of fermentation approaches to boost the nutritional value and biological activity of food products.

Investigations revealed that *Lactobacillus* fermentation strengthens the activation of pathways that contribute to carbohydrate decomposition, including glycolysis and the pathway of pentose phosphate [71]. Additionally, the fermentation process carried out by *Lactobacillus* strains results in the synthesis of short-chain fatty acids (SCFAs) via the disintegration of complex carbohydrates. This eventually impacts the pathways that regulate the metabolism of SCFA and, thus, energy production [72]. Furthermore, the fermentation of PKM by *Lactobacillus* influences the pathways that govern the metabolism of amino acids, involving either their synthesis or breakdown, which is in agreement with the findings of our experiment. This process could possibly boost the nutritional value of the fermented item by augmenting the accessibility of key amino acids and enhancing overall protein quality [73].

*Lactobacillus* fermentation of PKM contributes to pathways associated with the synthesis of bioactive substances and vitamins. In particular, this fermentation strengthens the enrichment of pathways that regulate the synthesis of B vitamins, such as riboflavin and folate, which are vital for multiple processes in cells involving the utilization of energy and nucleic acid production [74]. Furthermore, bioactive molecules developed throughout *Lactobacillus* fermentation, which include antioxidants and peptides that inhibit microbes, could impact pathways related to oxidative stress reactions and immune regulation. This might improve the functional properties of the fermented item, as demonstrated by our present study [75,76].

We developed a metagenomics assessment to evaluate the microbial communities that occur in the process of fermentation of PKM. The findings of our research demonstrated the presence of an extensive number of microbial classes, which comprise lactic acid-producing bacteria, that are known for their potential to create antioxidant peptides via distinct metabolic pathways. Soriano et al. [77] and Mamuad et al. [78] studied the influence of *Lactobacillus* fermentation on microbial biodiversity in grains. This research demonstrated microbial community modifications, including increased populations in LAB such as *Lactobacillales*. These results correspond with our microbial community barplot and the network evaluation of PMEL, indicating that most of the bacteria fall under *Lactobacillales* and contribute about 94% of the overall bacterial community. Multiple factors lead to the multiplication of LAB throughout *Lactobacillus* fermentation. First of all, *Lactobacillus* species make lactic acid as a consequence of their metabolic processes, which provides an acidic atmosphere by decreasing the pH value that favors their growth [79]. More importantly, these bacteria typically go beyond other species of bacteria in concurrence by successfully utilizing fermentable sugars that exist in the substrate [80]. In the end, the mechanism of *Lactobacillus* fermentation might end up in the development of antibiotic compounds that impede the growth of other microorganisms, thereby promoting the predominance of LAB [26,81].

*Lactobacillus* fermentation serves an important part in modifying the metabolite profile and microbiome of PKM, predominantly through enzymatic reactions that break down intricate polysaccharides, protein molecules, and fatty substances. During fermentation, *Lactobacillus* species contribute to multiple enzymes, such as glycoside hydrolases, proteases, and lipases, which deteriorate the fiber, protein, and fat constituents of PKM into less complicated molecules like monosaccharides, peptides, and fatty acids. This enzyme digestion not only enhances the nutritional importance of PKM by improving the bioavailability of nutrients but also boosts the release of bioactive components, including phenolics and flavonoids, which have been recognized for their antioxidant characteristics [82,83]. Furthermore, fermentation by *Lactobacillus* may promote the synthesis of organic acids such as lactic acid, which reduces the pH and hinders the proliferation of spoilage microbes, thereby enhancing the shelf life and safety of the food item [84]. This modification in pH assists in deliberately strengthening the populations of fermentative microbes, thereby enhancing the whole microbiome composition [85]. The metabolic switches resulting from *Lactobacillus* fermentation led to an altered metabolite profile that increases the functional and nutritional attributes of PKM, resulting in a more advantageous component in animal feed as well as applications involving biotechnology.

### 4.2. Correlation Evaluation of Microbial Population with Antioxidant Biopeptides and CAZy

We revealed the complicated interaction between the metabolism of microbes and the synthesis of bioactive compounds in fermented PKM by incorporating metabolomics and metagenomics results. Our investigation discovered different metabolite–microbe relationships, emphasizing critical metabolic processes and enzyme expression necessary for the generation of antioxidant compounds. Moreover, Spearman correlation studies uncovered the probable synergistic relationship between metabolites and microbial taxa, offering a mechanistic understanding of the antioxidant potential of the fermenting technique.

During fermentation, *L. plantarum* could promote the excretion and modification of flavonoids through enzymatic reactions, catalysing the disintegration of complex polyphenols into less complicated forms that are easier to absorb by the body. This enzymatic activity improves the bioavailability of flavonoids, possibly enhancing their physiological advantages [86]. Previous studies have illustrated that *L. plantarum* has a close connection with certain flavonoids like daidzein and kaempferol and their by-products, vanillic acid and genistin. The fermentation procedure involving LAB elevated the overall quantity of flavonoids and transformed the structure of certain ones, leading to improved antioxidant properties and enhanced endurance against tyrosinase [87]. The fermentation of kiwifruit by employing *L. plantarum* demonstrated a positive effect on its phenolic characteristics by increasing the concentrations of compounds, including dihydroxy coumarin, particularly protocatechuic acid. This enhancement inevitably strengthened the antioxidant and scavenging capacities of the processed kiwifruit [88]. These modifications in the phenolic substances may be attributed to the glucosidase enzyme generated by *L. plantarum*, which leads to alterations in the chemical structure of these molecules [89]. Feruloyl esterase enzymes assist in dispensing ferulic acid or p-coumarate from embedded phenols. L. plantarum yields a type of phenolic acid decarboxylase peptide, which influences these phenolic molecules and contributes to the overall antioxidant effectiveness [90]. Our correlation study findings revealed significant associations between the occurrence of LAB and higher levels of specific flavonoids, demonstrating an obvious connection between microbial development and the elevation of flavonoids.

*Lactobacillus* fermentation influences the profile of carbohydrate-active enzymes (CAZy), strengthening the decomposition of complex carbohydrates and boosting the absorption and digestion of essential nutrients [91]. Investigations on the association between species and CAZy enzymes offered a comprehensive understanding of the wide range of enzymes utilized by *Lactobacillus* during the process of fermentation, including glycoside hydrolases, polysaccharide lyases, carbohydrate esterases, and glycosyl transferases. These enzymes are essential in breaking down complicated carbohydrates, such as plant cell wall polymers, into simpler digestible sugars like glucose and galactose. The treatment leads to the creation of several different fermentation products, including organic acids and bioactive molecules, which strengthen the sensory qualities and dietary benefits of fermented items [92,93,94]. The most recent studies illustrate that specific CAZy communities exhibit a strong connection to the metabolic activities of *Lactobacillus* species, amending their abilities to break down intricate polysaccharides into simpler carbohydrates [95]. The elevated level of glycoside hydrolases appears to be linked to the higher concentration of *L. plantarum* during the process of fermenting, implying a beneficial association that enhances the productivity of the fermentation procedure [96]. In the present research, multiple CAZy enzymes have been identified to interact strongly with bacterial species, involving carbohydrate esterases, glycoside hydrolases, polysaccharide lyases, and glycosyl transferases. These enzymes may have accounted for the increased nutritional content and antioxidant potency of PMEL.

### 4.3. Polyphenolic and Flavonoid Content and Antioxidant Capability

The LAB, primarily *Lactobacillus* spp., produces metabolites that consist of exopolysaccharides, phenolic molecules, and organic acids. These substances offer the cornerstone for the fermented item’s antioxidant benefits [97]. The findings of our experiment demonstrate that these compounds, which contain antioxidant properties, considerably boost the fermented item’s antioxidant activity by effectively counteracting harmful unbound radicals and minimizing oxidative-related stress.

Flavonoids and polyphenols are metabolically active compounds that are renowned for their antioxidant and beneficial health capabilities [98]. Investigations have demonstrated that *Lactobacillus* fermentation could lead to a considerable increase in both flavonoid and polyphenol contents of fermented food [99]. Meng et al. [86] performed a study in which they revealed that a procedure of fermentation employing *Lactobacillus* contributed to a massive increase in the flavonoid composition of loquat juice that finally resulted in enhanced antioxidant capabilities. Verni et al. [100] discovered a high elevation in the polyphenol and flavonoid content and antioxidant capacity of grains after it was fermented using *Lactobacillus plantarum*.

The scavenging proportion of PMEL was enhanced following *Lactobacillus* fermentation. *Lactobacillus* bacteria exhibited enzymatic attributes that may degrade complex compounds present in PKM into simpler molecules, thereby boosting the availability of bioactive compounds [68,101]. Moreover, *Lactobacillus* fermentation might end up in the production and dispersion of antioxidant compounds through metabolic mechanisms involving the alteration of phenolic intermediates into bioactive substances [102]. Furthermore, the method of fermentation provides an acidic environment that promotes the retrieval and accessibility of bioactive compounds, therefore enhancing their level of concentration in the fermented food [68]. These could potentially be the foundations of PMEL’s improving scavenging capabilities.

Modifications in the composition of unprocessed palm kernel meal, especially fiber composition and nutritional value, might influence fermentation accuracy and the production of antioxidant peptides, possibly restricting the applicability of the study’s findings. These variations were not fully accounted for, which might impact the coherence of metabolomic and metagenomic outcomes. The variation in nutritional and fiber content can affect microbial growth, which in turn can affect the whole microbiome composition and metabolite production. The high-fat level in certain PKM batches may restrict microbial growth, as *Lactobacillus* strains opt for carbohydrate-rich materials. Additionally, the excessive lignocellulosic fiber percentage of PKM exhibits a further challenge, as these fibers are not digested readily by the microbes. Variation in fiber levels among various PKM batches lessens the accessibility of fermentable sugars, which impedes fermentation efficacy [15,25,103]. This study concentrated primarily on specific strains of *Lactobacillus*, probably overlooking contributions from other communities of microbes. Fermentation is a complicated procedure usually driven by a consortium of microorganisms, each incorporating various enzymes and metabolic processes. By emphasizing primarily *Lactobacillus*, the study likely neglected other microbial populations (such as yeasts, fungi, or other bacteria) that might improve antioxidant peptide biosynthesis [104]. Some low-abundance metabolites might have been neglected due to detection sensitivity. These metabolites, although present in smaller quantities, may contribute to the overall bioactivity and functionality of the fermentation process. The research might depend predominantly on in vitro or computational approaches, and without verification in vivo, it restricts the knowledge of the real-world performance and bioavailability of the newly identified antioxidant peptides. The variety of microbial strains employed in this research could be limited, which could fail to adequately represent the potential diversity of peptide production pathways or wider ecological relationships that could take place in more diverse microbial environments.

## 5. Conclusions

In conclusion, the combined application of metabolome and metagenome analyses has effectively unveiled the biosynthesis pathways of novel peptides with antioxidant properties in the fermented PKM. This study revealed the potential of *Lactobacillus* fermentation to improve the bioactive functions of PKM by facilitating the synthesis and release of antioxidant peptides via the enzymatic breakdown of complicated carbohydrates and lignocellulosic structures. The outcomes offer valuable insights into the underlying metabolic pathways and provide a foundation to enhance the nutritional and functional benefits of PKM. The results demonstrate the enormous potential of microbial fermentation in valorizing PKM, transforming it into an efficient food component with enriched antioxidant properties. The research not only broadens the understanding of microbial transformation mechanisms on lignocellulosic substrates but also unlocks new avenues toward creating bioactive substances through sustainable biotechnological methods. However, further in vivo testing and study of different bacterial strains and palm kernel meal sources is essential for fully comprehending the feasible applications of these bioactive substances in the animal nutrition and food industry. Furthermore, extending the diversity of bacterial strains employed during the fermentation process could assist in exploring a broader range of metabolic processes for novel antioxidant peptide production. Scaling up the fermentation technique for industrial application while improving conditions for optimal yield is also crucial. Future studies ought to explore the practical uses of these peptides in the food and pharmaceutical industry to evaluate their efficacy as health-promoting ingredients. Conclusively, carrying out long-term stability research studies might guarantee the antioxidant peptides preserve their efficacy during commercial manufacturing and delivery.

## Data Availability

Data will be available upon request.

## Supplementary Materials

The following supporting information can be downloaded at: https://www.mdpi.com/article/10.3390/antiox13101253/s1, Figure S1. (A) Sample clustering dendrogram; (B) Metabolites dendrogram; (C) Correlation between modules; and (D) Module clustering dendrogram.
