# Supplementary material for: Metabolome and Metagenome Integration Unveiled Synthesis Pathways of Novel Antioxidant Peptides in Fermented Lignocellulosic Biomass of Palm Kernel Meal
Source: Antioxidants (Basel). 2024 Oct 17;13(10):1253. doi: 10.3390/antiox13101253 (PMC11505245; doi:10.3390/antiox13101253)
Supplement: Supplementary file 1 [file antioxidants-13-01253-s001.zip › antioxidants-3253049-supplementary.pdf]

Supplementary Data

The sample cluster dendrogram revealed that there was no outlier in the model (Fig. 8A). A total of 864 DEMs were selected and grouped into four modules (MEblue, MEbrown, MEturquoise and MEyellow), and the metabolites that failed to fall under any module were placed in the grey module and were not considered in the later analysis (Fig. 8B). The relationship between modules via correlation is showed in (Fig. 8C) and via dendrogram is showed in (Fig. 8D). The module clustering dendrogram revealed that MEyellow is closely related to MEturquoise. The correlation between modules depicted that MEblue and MEbrown possessed close relationships whereas, MEturquoise and MEyellow were closely associated.

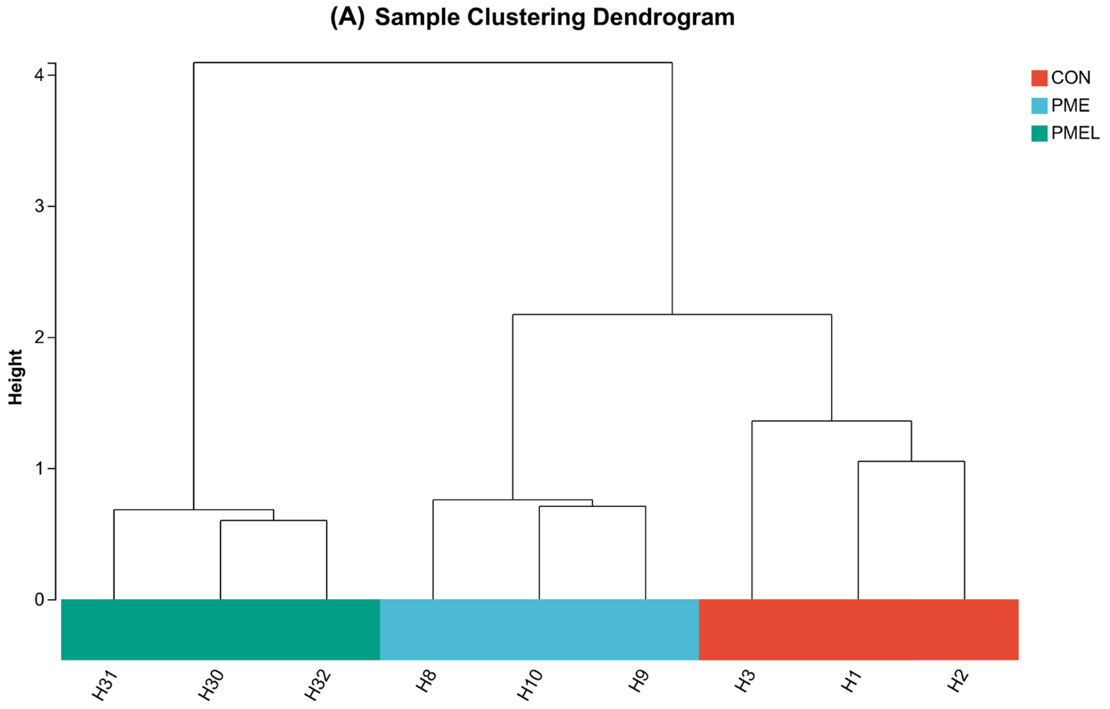

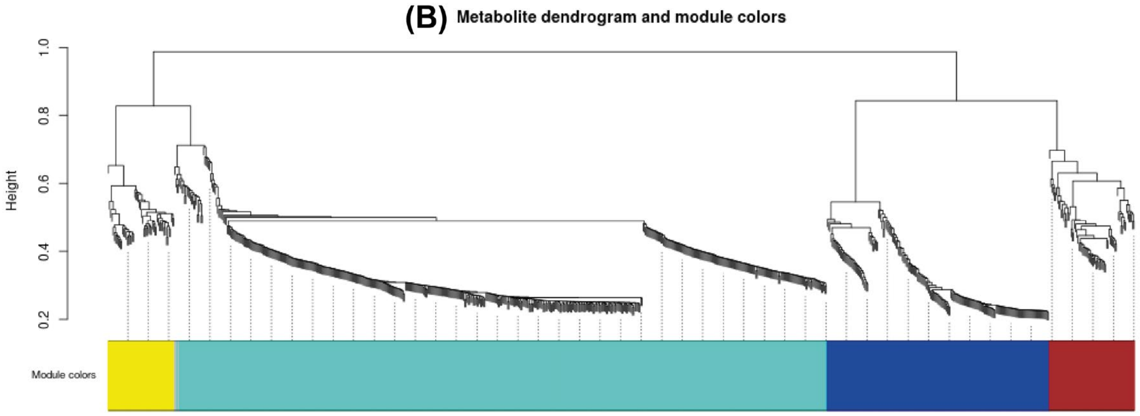

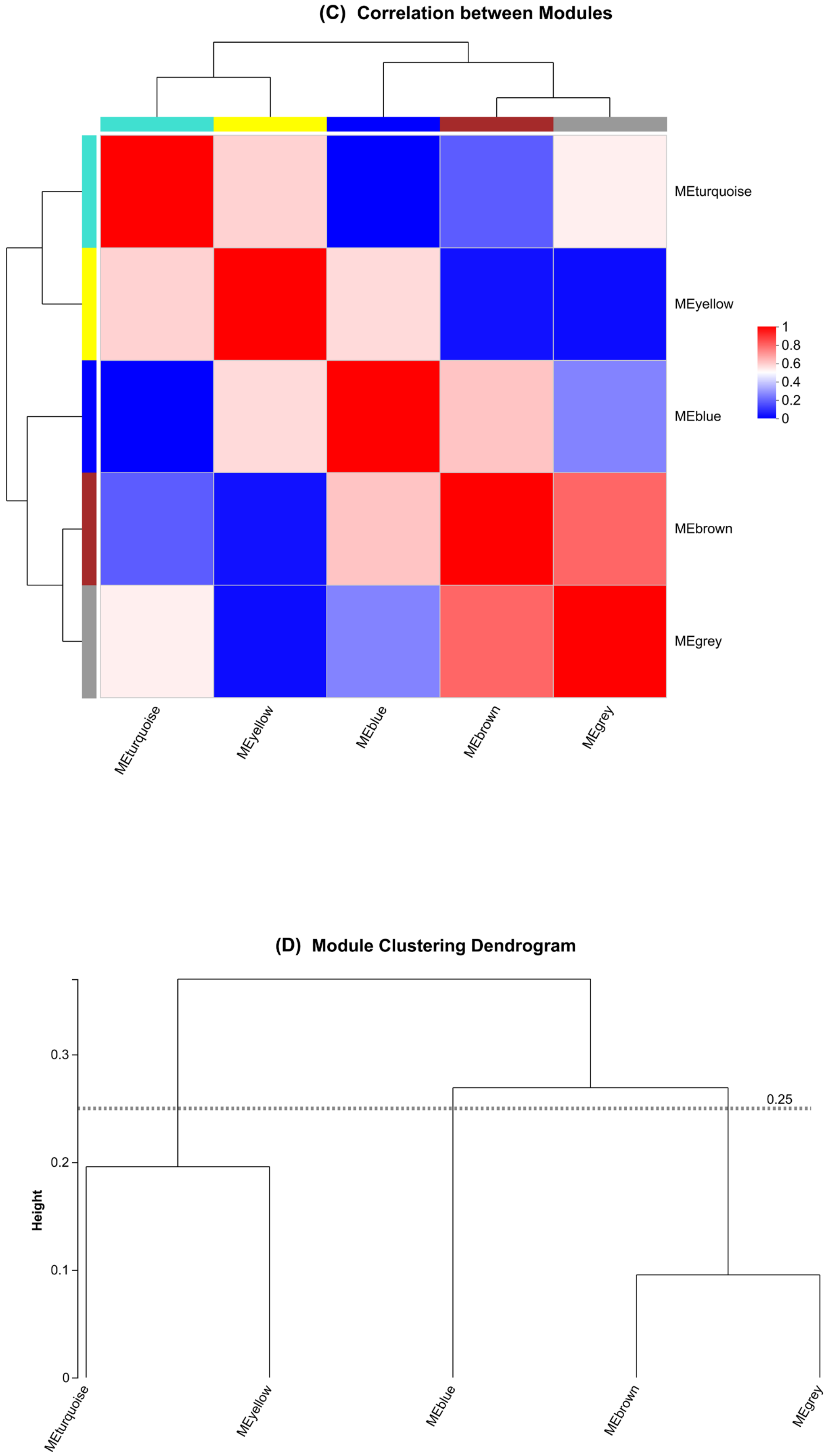

Fig 1S (A) Sample clustering dendrogram; (B) Metabolites dendrogram; (C) Correlation between modules; and (D) Module clustering dendrogram.
